# The *Legionella* autoinducer LAI-1 is delivered by outer membrane vesicles to promote interbacterial and interkingdom signaling

**DOI:** 10.1016/j.jbc.2023.105376

**Published:** 2023-10-20

**Authors:** Mingzhen Fan, Patrick Kiefer, Paul Charki, Christian Hedberg, Jürgen Seibel, Julia A. Vorholt, Hubert Hilbi

**Affiliations:** 1 Institute of Medical Microbiology, University of Zürich, Zürich, Switzerland; 2 Institute of Microbiology, ETH Zürich, Zürich, Switzerland; 3 Institute of Organic Chemistry, University of Würzburg, Würzburg, Germany; 4 Institute of Chemistry and Umeå Center for Microbial Research, Umeå University, Umeå, Sweden

**Keywords:** α-hydroxyketone, autoinducer, amoeba, cell-cell communication, *Dictyostelium*, host-pathogen interaction, interkingdom signaling, *Legionella*, macrophage, outer membrane vesicle, quorum sensing

## Abstract

*Legionella pneumophila* is an environmental bacterium, which replicates in amoeba but also in macrophages, and causes a life-threatening pneumonia called Legionnaires’ disease. The opportunistic pathogen employs the α-hydroxy-ketone compound *Legionella* autoinducer-1 (LAI-1) for intraspecies and interkingdom signaling. LAI-1 is produced by the autoinducer synthase *Legionella* quorum sensing A (LqsA), but it is not known, how LAI-1 is released by the pathogen. Here, we use a *Vibrio cholerae* luminescence reporter strain and liquid chromatography-tandem mass spectrometry to detect bacteria-produced and synthetic LAI-1. Ectopic production of LqsA in *Escherichia coli* generated LAI-1, which partitions to outer membrane vesicles (OMVs) and increases OMV size. These *E. coli* OMVs trigger luminescence of the *V. cholerae* reporter strain and inhibit the migration of *Dictyostelium discoideum* amoeba. Overexpression of *lqsA* in *L.pneumophila* under the control of strong stationary phase promoters (P_*flaA*_ or P_*6SRNA*_), but not under control of its endogenous promoter (P_*lqsA*_), produces LAI-1, which is detected in purified OMVs. These *L*. *pneumophila* OMVs trigger luminescence of the *Vibrio* reporter strain and inhibit *D. discoideum* migration. *L. pneumophila* OMVs are smaller upon overexpression of *lqsA* or upon addition of LAI-1 to growing bacteria, and therefore, LqsA affects OMV production. The overexpression of *lqsA* but not a catalytically inactive mutant promotes intracellular replication of *L. pneumophila* in macrophages, indicating that intracellularly produced LA1-1 modulates the interaction in favor of the pathogen. Taken together, we provide evidence that *L. pneumophila* LAI-1 is secreted through OMVs and promotes interbacterial communication and interactions with eukaryotic host cells.

The gram-negative bacterium *Legionella pneumophila* is an opportunistic pathogen, which upon inhalation of bacteria-laden aerosols replicates in lung macrophages and causes a life-threatening pneumonia termed Legionnaires’ disease (1, 2, 3). In the environment, *L. pneumophila* persists and replicates in free-living protozoa (4, 5, 6). Intriguingly, *L. pneumophila* subverts the bactericidal potential of macrophages and amoeba in a similar manner and within these evolutionary distant phagocytes forms an endoplasmic reticulum-associated replication-permissive compartment called the *Legionella*-containing vacuole (LCV) (7, 8, 9, 10). To govern LCV formation, *L. pneumophila* employs the intracellular multiplication/defective organelle trafficking (Icm/Dot) type IV secretion system, which translocates more than 300 “effector” proteins into eukaryotic host cells, where they subvert various processes, including trafficking pathways, cytoskeleton dynamics, signal transduction, and metabolism (9, 11, 12, 13, 14, 15, 16).

*L. pneumophila* employs the *Legionella* quorum sensing (Lqs) system for small molecule intraspecies and interkingdom signaling (Fig. 1) (17, 18). The quorum sensing system is rather broadly distributed among environmental bacteria, including the families Legionellaceae, Vibrionaceae, Burkholderiaceae, Chlorobiaceae, and Oxalobacteraceae (17, 19), and fairly conserved among *Legionella* spp.: 19 out of 58 species harbor a complete *lqs* cluster (20).

**Figure 1:**
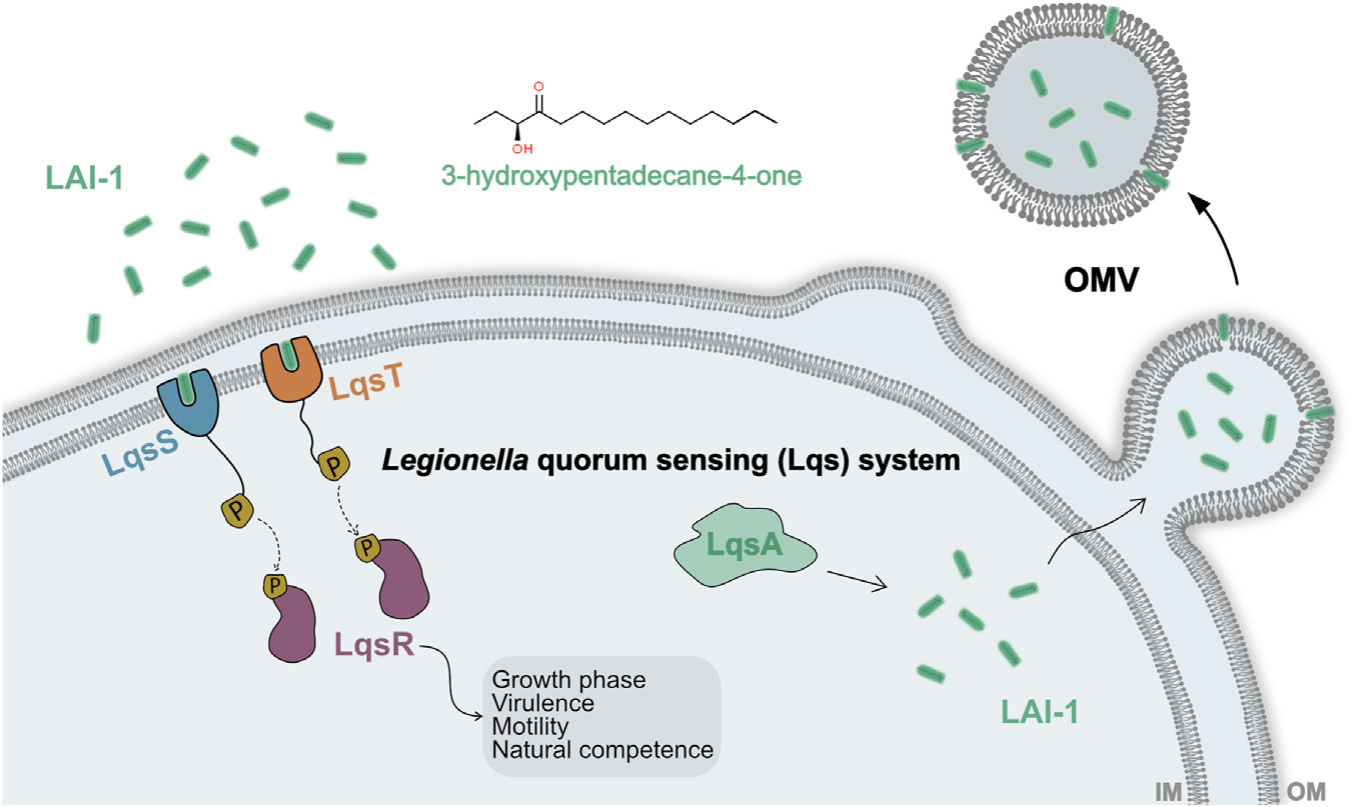
**The *Legionella pneumophila* Lqs system:production, detection, and release of LAI-1.** The Lqs (*Legionella* quorum sensing) system produces, detects, and responds to the small signaling molecule LAI-1 (*Legionella* autoinducer-1, 3-hydroxypentadecane-4-one). The system comprises the autoinducer synthase LqsA, the cognate membrane-bound sensor kinases LqsS and LqsT, and the prototypic response regulator LqsR. LAI-1 produced by LqsA partitions into outer membrane vesicles (OMVs), through which the hydrophobic compound is released from the bacteria to promote interbacterial as well as interkingdom signaling.

The Lqs system includes the pyridoxal-5′-phosphate-dependent autoinducer synthase LqsA (21), which is 41% identical to *Vibrio cholerae* CqsA (22, 23). Furthermore, the system comprises the homologous sensor histidine kinases LqsS (24) and LqsT (25), and the cognate response regulator LqsR (26, 27), which dimerizes upon phosphorylation (28, 29). LqsS negatively regulates a pleiotropic transcription factor termed *Legionella* virulence and biofilm regulator (LvbR), which controls *L. pneumophila* virulence, biofilm architecture, and natural competence for DNA uptake (30). LvbR also regulates the nitric oxide (NO) sensor and di-guanylate cyclase inhibitor Hnox1, and thus, positively regulates the production of the second messenger cyclic di-guanosine monophosphate (c-di-GMP). Accordingly, quorum sensing is linked to c-di-GMP signaling in *L. pneumophila* (30, 31).

The Lqs system produces, detects, and responds to the low molecular weight compound *Legionella* autoinducer-1 (LAI-1, 3-hydroxypentadecane-4-one) (17, 32) (Fig. 1). The quorum sensing system and synthetic LAI-1 regulate various traits of *L. pneumophila*, such as motility and flagellum production (33), virulence (18, 34), the bacterial growth phase switch and temperature-dependent culture density (26, 35), expression of a “fitness island”, and natural competence for DNA uptake (18, 34). Moreover, the Lqs-LvbR signaling network regulates the migration of *Acanthamoeba castellanii* amoeba through *L. pneumophila* biofilms (36), as well as—upon exposure of *Dictyostelium discoideum* amoeba, macrophages, or epithelial cells to *L. pneumophila*—the motility of eukaryotic cells (37). LAI-1 is rather hydrophobic and might partition into the aqueous or lipid bacterial compartments (21, 33). It is not known how LAI-1 is secreted and delivered by *L. pneumophila*.

Gram-negative bacteria form and release so-called outer membrane vesicles (OMVs), which are spherical vesicles of ca. 10 to 300 nm diameter confined by a membrane bilayer with lipopolysaccharide in the outer leaflet (38, 39). OMVs are produced and released by planktonic, biofilm, and intracellular bacteria under different physiological conditions, and they transport proteins, hydrophobic small molecules, and nucleic acids between bacteria or between bacteria and eukaryotic cells (40, 41). OMVs adopt a plethora of functions, including disposal of “waste” material (proteins, lipids, and peptidoglycan), secretion of virulence factors (toxins, proteases, and lipases), nutrient scavenging (carbon sources, and micronutrients: *e.g.*, iron), inactivation of antibiotics, titration of phages, transport of signaling molecules, as well as delivery of DNA and regulatory RNA (42). While the production of OMVs comes with many advantages for the producing bacteria, it also comes with a price, as OMVs are complex and energetically costly to produce, and they may elicit adverse immune responses (43).

*L. pneumophila* produces OMVs, which are enriched in virulence-relevant proteins such as proteolytic and lipolytic enzymes, the macrophage infectivity potentiator (Mip) protein, Icm/Dot components and substrates, as well as the major flagellum component flagellin (44, 45). These OMVs can fuse with eukaryotic membranes, but do not kill host cells and rather promote the growth of amoeba, activate mammalian cells, and modulate their cytokine response (44, 46, 47, 48). The host cell’s innate immune response is also targeted by small RNAs delivered by *L. pneumophila* OMVs (49). Moreover, *L. pneumophila* OMVs bind to and are internalized by macrophages (46, 50), where—independently of the Icm/Dot type IV secretion system—they inhibit phagosome-lysosome fusion (51) and promote intracellular bacterial replication at later time points of infection (47).

In this study, we tested the hypothesis that the *L. pneumophila* signaling compound LAI-1 partitions to OMVs and is secreted by these vesicles. We provide evidence that LAI-1 partitions to and is secreted by OMVs formed in either *Escherichia coli* or *L. pneumophila* overexpressing the autoinducer synthase gene *lqsA*. These OMVs mediate intrabacterial as well as interkingdom communication. Finally, the overexpression of *lqsA*, but not a catalytically inactive form, promotes intracellular replication of *L. pneumophila* in macrophages.

## Results

### LAI-1 detection by a *Vibrio* reporter strain and LC-MS/MS

**Figure 2:**
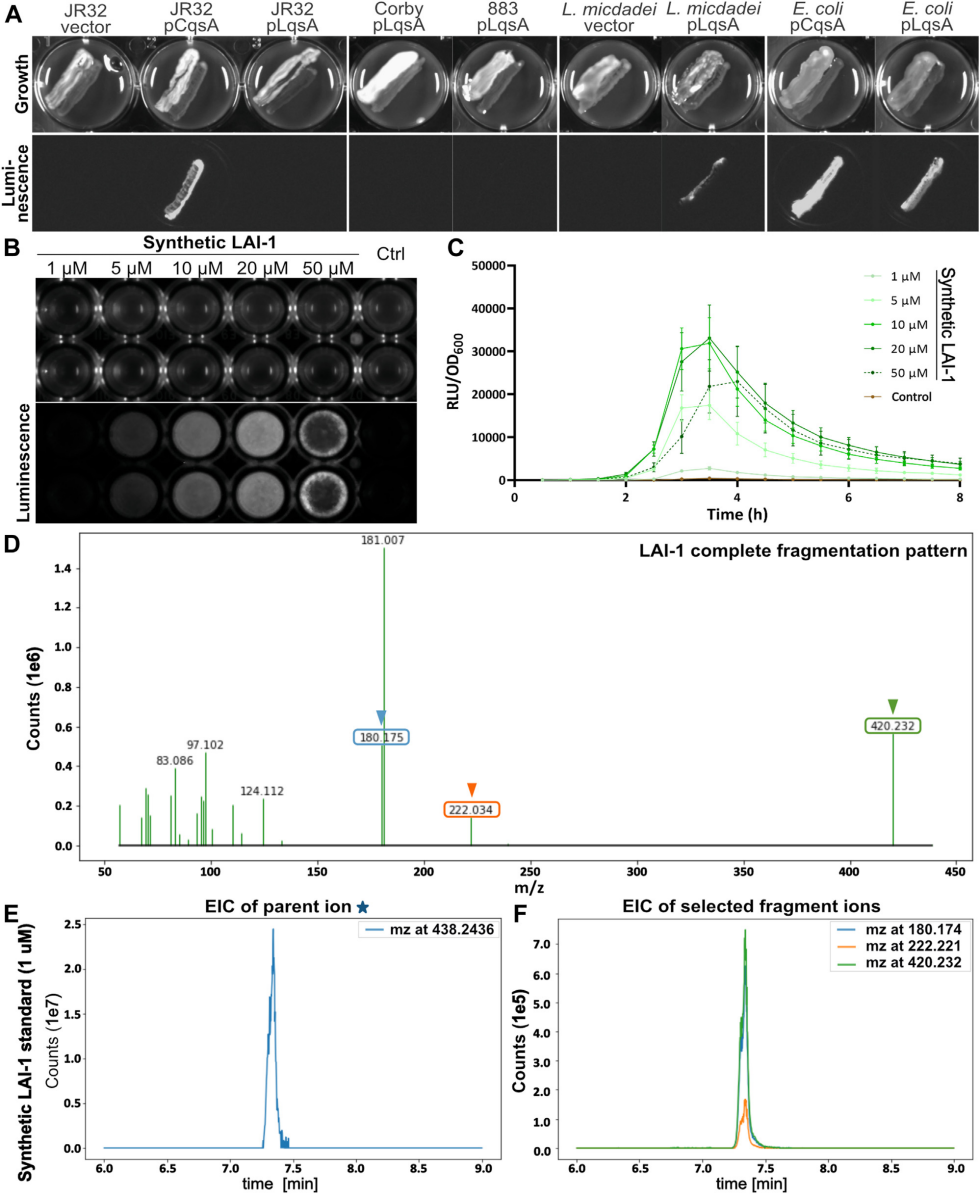
**LAI-1 detection by *Vibrio cholerae* MM920 and LC-MS/MS.***A*–*C*, luminescence of the *V. cholerae* α-hydroxyketone reporter strain MM920. *A*, *Legionella pneumophila* strains (JR32, Corby, clinical isolate 883), *Legionella micdadei*, or *Escherichia coli* harboring an empty vector (pTS-10) or plasmids expressing *lqsA* (pTS-2) or *cqsA* (pTS-6) under control of the P_*tac*_ promoter were grown on CYE agar in 24 well plates for 2 days, before the *V. cholerae* reporter strain MM920 was streaked out in parallel. After another day, growth was assessed (*upper panels*), and bioluminescence was determined with a luminometer (*lower panels*). *V. cholerae* MM920 was treated with the concentrations of synthetic LAI-1 indicated, DMSO was included as negative control, and luminescence intensity was (*B*) visualized by a gel documentation system (15 min exposure time), or (*C*) measured by a plate reader (30 °C, 8 h). *D*–*F*, MS fragment spectra of LAI-1. *D*, the fragment ions at m/z 180.174 and 222.221 cover the complete structure of LAI-1 and exhibit high specificity. For detection of LAI-1, the focus was placed on fragment ions at m/z 180.174 (*light blue arrowhead*), 222.221 (*orange arrowhead*), and 420.232 (*green arrowhead*). Positive detection and identification results were obtained from the extracted ion chromatogram (EIC) of (*E*) the parent ion at m/z 438.24, along with (*F*) compound-specific fragment ions at m/z 180.174 (*light blue line*), 222.221 (*orange line*), and 420.232 (*green line*). LC-MS/MS analysis was performed with 1 μM synthetic LAI-1 as a standard. The data shown are means and standard deviations of technical triplicates (*C*) and representative of three independent experiments. CYE, charcoal yeast extract; DMSO, dimethyl sulfoxide; LAI-1, *Legionella* autoinducer-1; LC-MS/MS, liquid chromatography-tandem mass spectrometry; Lqs, *Legionella* quorum sensing; RLU, relative light units.

The *L*. *pneumophila* signaling compound, LAI-1, has been identified as 3-hydroxypentadecane-4-one by liquid chromatography-tandem mass spectrometry (LC-MS/MS) upon overexpression of *lqsA* controlled by the P_*tac*_ promoter in *E. coli* and *L. pneumophila* (21). Under the conditions tested, *L. pneumophila* strains did not produce endogenous LAI-1 detectable by mass spectrometry (MS) or by the *V. cholerae* α-hydroxyketone luminescence reporter strain MM920 (21) (Fig. 2*A*). Upon P_*tac*_-controlled overexpression of *lqsA* in *E. coli*, *Legionella micdadei* or *L. pneumophila* growing on charcoal yeast extract agar plates, the *V. cholerae* strain MM920 detected a signal produced by *E. coli* and *L. micdadei*, but not by several *L. pneumophila* strains (JR32, Corby, clinical isolate #883) (Fig. 2*A*). However, upon growing in N-(2-acetamido)-2-aminoethanesulfonic acid-buffered yeast extract (AYE) broth, *L. pneumophila* overexpressing *lqsA* produced a signal, which was detected by the *V. cholerae* reporter strain in culture supernatants, albeit only during a short period of time during growth (Fig. S1). Contrarily, the signal produced by *L. pneumophila* overexpressing *cqsA* was robustly detected by the *V. cholerae* reporter strain, regardless of whether *L. pneumophila* was grown on agar plates or in broth (Figs. 2*A* and S1).

Given the apparently low amounts of LAI-1 produced by *L. pneumophila*, we next tested whether and with which sensitivity the *V. cholerae* reporter strain detects LAI-1. To this end, 1 to 50 μM synthetic LAI-1 was added to the reporter strain and luminescence was measured (Fig. 2, *B* and *C*). 1 to 20 μM synthetic LAI-1 triggered the luminescence response by the *V. cholerae* reporter strain in a dose-dependent manner, indicating that the reporter strain indeed recognizes the *L. pneumophila* signaling molecule. Fifty micromolar of LAI-1 interfered with the growth of *V. cholerae*, and accordingly, the luminescence emitted by the reporter strain was reduced. The detection limit for LAI-1 of the *V. cholerae* luminescence reporter assay was ca. 1 μM (Fig. 2*C*).

Synthetic LAI-1 was also detected by LC-MS/MS (Figs. 2, *D*–*F* and S2). After oximation with O-(2,3,4,5,6-pentafluorobenzyl) hydroxylamine hydrochloride (O-PFB), a standard of 1 μM LAI-1 was analyzed. LAI-1 was detected and identified by the extracted ion chromatogram, showing the parent ion at m/z 438.24 (Figs. 2*E* and S2), along with compound-specific fragment ions at m/z 180.174, 222.221, and 420.232 (Figs. 2*F* and S2). The detection limit of the LC-MS/MS approach for LAI-1 was ca. 10 fmol corresponding to 1 μl of a 10 nM standard solution.

### LAI-1 partitions to OMVs of *E. coli* ectopically expressing *lqsA*

**Figure 3:**
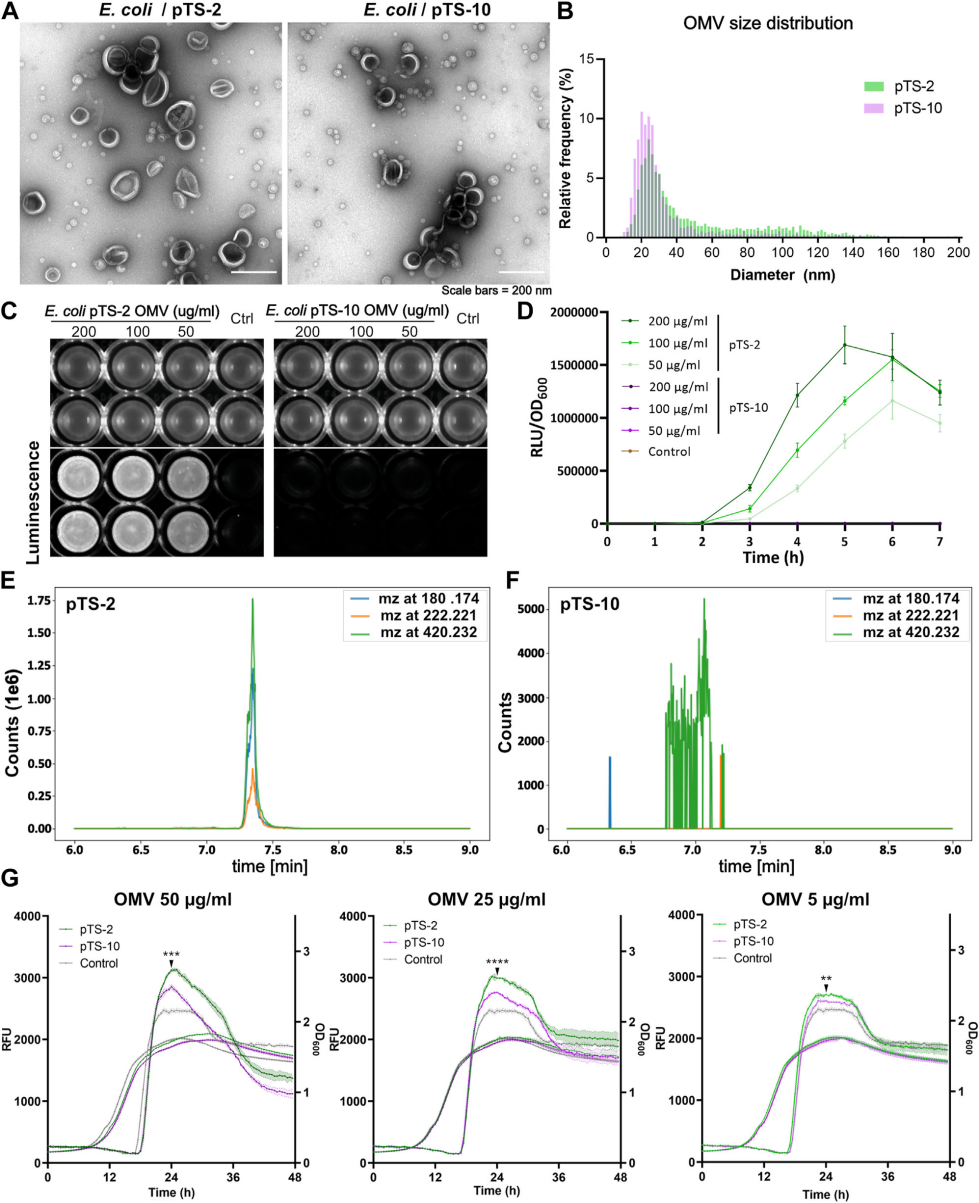
**LAI-1 partitions to OMVs of *Escherichia coli* ectopically expressing *lqsA* and promotes interbacterial signaling.** OMVs of *E. coli* TOP10 harboring pTS-2 (P_tac_-*lqsA*) or pTS-10 (empty vector) were isolated from overnight cultures induced with 1 mM IPTG for 4 h. *A*, negative staining transmission electron microscopy (TEM) images of OMVs derived from *E. coli* harboring pTS-2 (*left*) or pTS-10 (*right*). *B*, size distribution analysis of OMVs of *E. coli* pTS-2 or pTS-10. *C* and *D*, luminescence of *Vibrio cholerae* MM920 mixed with OMVs from *E. coli* harboring pTS-2 or pTS-10 of the protein concentrations indicated (control: DMSO). Intensity was (*C*) visualized by a gel documentation system (15 min exposure time), or (*D*) measured by a plate reader (30 °C, 7 h). *E* and *F*, LC-MS/MS analysis of OMVs from *E. coli* pTS-2 or pTS-10 (1 mg protein). Extracted ion chromatograms (EICs) of fragment ions at m/z 180.174 (*light blue*), 222.221 (*orange*), and 420.232 (*green*) indicated the presence of LAI-1 in OMVs of *E. coli* pTS-2 (P_*tac*_*-lqsA*) but not in OMVs of *E. coli* pTS-10. *G*, *L. pneumophila* expressing P_*flaA*_-*gfp* was treated with OMVs of *E. coli* harboring pTS-2 (P_*tac*_*-lqsA*) or pTS-10 (empty vector). OMVs from *E. coli* pTS-2 induced the expression of *gfp* under the control of P_*flaA*_ in a dose-dependent manner. The data shown are means and standard deviations of technical triplicates (*D* and *G*) ∗∗*p* ≤ 0.01; ∗∗∗*p* ≤ 0.001; ∗∗∗∗*p* ≤ 0.0001 and representative of three independent experiments. DMSO, dimethyl sulfoxide; LAI-1, *Legionella* autoinducer-1; LC-MS/MS, liquid chromatography-tandem mass spectrometry; Lqs, *Legionella* quorum sensing; OMV, outer membrane vesicle; RLU, relative light units.

To assess whether LAI-1 affects OMV formation and/or partitions into OMVs, we ectopically expressed *lqsA* in *E. coli* under the control of the P_*tac*_ promoter. OMVs were isolated from bacterial culture supernatants and enriched by ultracentrifugation as outlined in the Experimental procedures section (Fig. 3*A*). While *E. coli* expressing *lqsA* produced an OMV population ranging from approximately 10 to 140 nm in diameter, the control strain produced a population of OMVs, the majority of which ranged from 10 to 60 nm in diameter (Fig. 3*B*). Hence, the ectopic production of LqsA in *E. coli* seems to increase the average OMV size and thus to possibly affect the formation of OMVs.

We then exposed the *V. cholerae* reporter strain MM920 to OMV preparations purified from *E. coli* expressing *lqsA* or not (Fig. 3*C*). OMV preparations (50–200 μg protein/ml) purified from *E. coli* expressing *lqsA* resulted in a dose-dependent luminescence signal, while OMV samples prepared from the *E. coli* control strain or untreated reporter strain did not produce any luminescence (Fig. 3*D*). LAI-1 was also detected by LC-MS/MS in OMV preparations from *E. coli* expressing *lqsA* (Fig. 3*E*) but not from the *E. coli* control strain (Fig. 3*F*, Table 1).

**Table 1:** Detection of LAI-1 in OMV samples by LC-MS/MS

Sample	LAI-1 yield (nM)
Controls	
1 μM synthetic LAI-1	1155
PBS buffer	0
OMVs^a^	
*E. coli* pTS-2 (P_*tac*_-*lqsA*)	1778
*E. coli* pTS-10	0
JR32	0
Δ*lqsA*	0
JR32 pTS-2 (P_*tac*_-*lqsA*)	37
JR32 pTS-10	0
JR32 P_*lqsA*_*-lqsA*	10
JR32 P_*lqsA*_*-lqsA*^K258A^	0
JR32 P_*flaA*_*-lqsA*	140
JR32 P_*flaA*_*-lqsA*^K258A^	0
JR32 P_*6SRNA*_*-lqsA*	270
JR32 P_*6SRNA*_*-lqsA*^K258A^	0

a For each sample, 1 mg of OMVs (protein concentration) was processed and analyzed by LC-MS/MS as outlined in the Experimental procedures section. The values are representative for >2 measurements.

Previously, we have shown that synthetic LAI-1 at a concentration of 10 μM promotes the expression of P_*flaA*_-*gfp* and motility in *L. pneumophila* (33). Based on these findings, we sought to test whether OMVs purified from *E. coli* expressing *lqsA* also induced the expression of *gfp* under the control of P_*flaA*_ in *L. pneumophila*. Using this readout, OMV samples prepared from the *E. coli* strain producing LqsA induced the expression of P_*flaA*_-*gfp* in *L. pneumophila* in a dose-dependent manner (Fig. 3*G*). In contrast, OMVs from the *E. coli* strain harboring the empty plasmid barely induced the expression of P_*flaA*_-*gfp* in *L. pneumophila*. The expression of P_*flaA*_-*gfp* above background levels by OMVs from the *E. coli* strain harboring the empty plasmid is likely due to unidentified components of the *E. coli* OMVs. Accordingly, not only the *V. cholerae* reporter strain, but also *L. pneumophila* detects and responds to OMV-associated LAI-1. Taken together, the ectopic production of LqsA in *E. coli* produces LAI-1, which partitions to OMVs, affects OMV formation of the donor strain, and promotes interbacterial communication.

### *E. coli* OMVs containing LAI-1 inhibit the migration of *D. discoideum*

**Figure 4:**
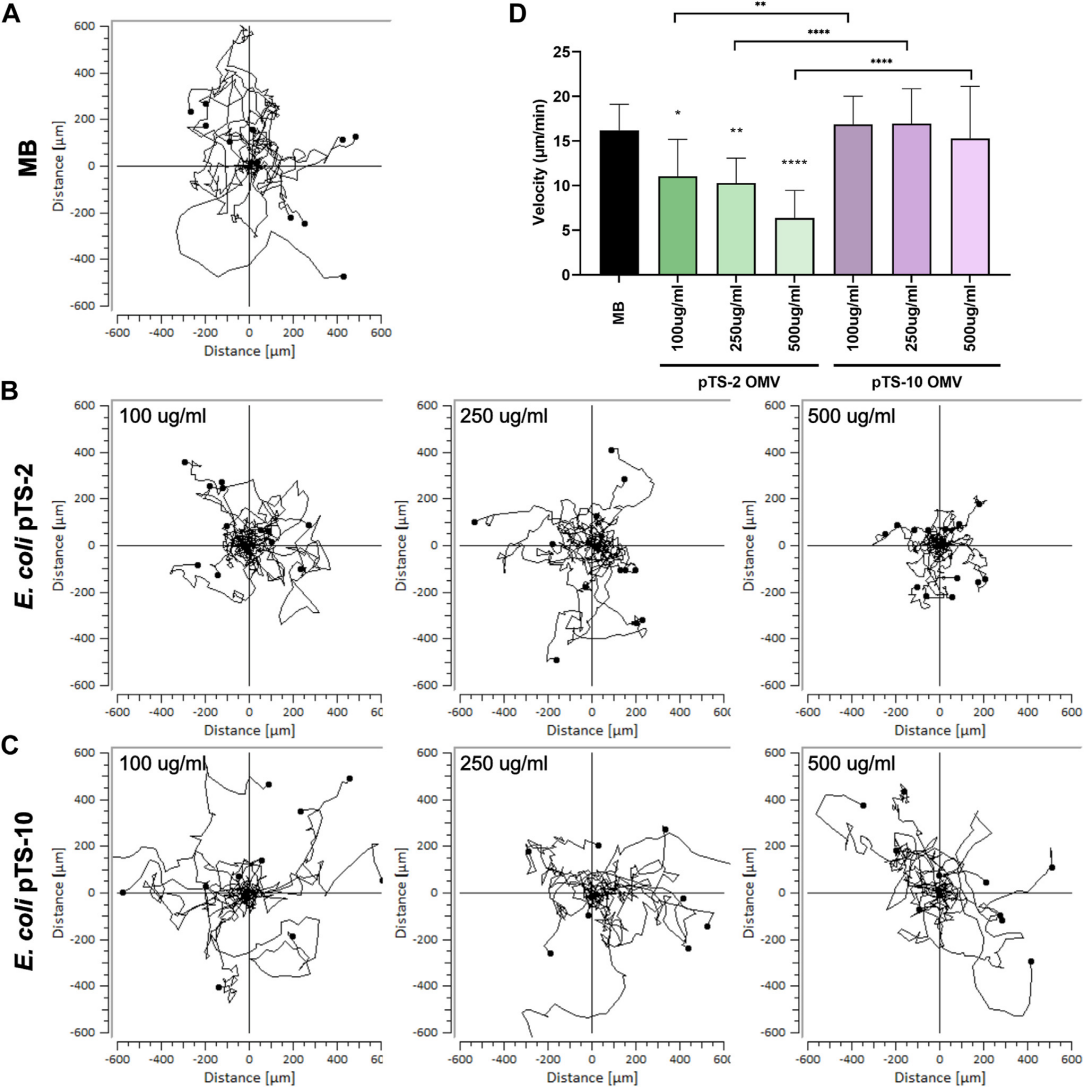
***Escherichia coli* OMVs harboring LAI-1 inhibit the migration of amoeba.***Dictyostelium discoideum* amoeba were treated with OMVs of *E. coli* TOP10 harboring pTS-2 (P_*tac*_-*lqsA*) or pTS-10 (empty vector) with a final protein concentration of 100 μg/ml, 250 μg/ml, or 500 μg/ml. For each sample, the migration of 10 to 15 amoeba was tracked over 2 h and the velocity was quantified. Migration trajectories of amoeba treated with (*A*) (2-(*N*-morpholino)ethanesulfonic acid)-buffered medium (control), (*B*) OMVs of *Escherichia coli* pTS-2, or (*C*) *E. coli* pTS-10. *D*, median of amoeba migration velocity. The data shown are velocity median and standard deviations of 10 to 15 amoeba per sample ∗*p* ≤ 0.1; ∗∗, *p* ≤ 0.01; ∗∗∗∗, *p* ≤ 0.0001 and representative of three independent experiments. LAI-1, *Legionella* autoinducer-1; Lqs, *Legionella* quorum sensing; OMVs, outer membrane vesicles.

The migration of *D. discoideum* is reduced by pretreatment with 10 μM synthetic LAI-1 (37) (Fig. S3). Based on this finding, we asked the question, whether *E. coli* OMVs containing LAI-1 would exhibit a comparable effect and promote interkingdom signaling. To this end, we treated *D. discoideum* amoeba with OMVs purified from *E. coli* expressing *lqsA* or not (Fig. 4). Compared to the (2-(*N*-morpholino)ethanesulfonic acid)-buffered (MB) medium control (Fig. 4*A*), the migration of the amoeba was inhibited in a dose-dependent manner by OMVs purified from *E. coli* expressing *lqsA* (Fig. 4*B*), but not by OMVs from *E. coli* harboring an empty plasmid (Fig. 4*C*). At the highest OMV concentration used (500 μg/ml protein), the velocity of the amoeba was reduced ca. 3-fold compared to the controls (Fig. 4*D*). Accordingly, OMV-associated LAI-1 not only promotes interbacterial communication but also interkingdom signaling.

### *L pneumophila* OMV production is regulated by LqsA and LAI-1

Similar to *E. coli* (Fig. 3*B*), the production of OMVs by *L. pneumophila* might be affected by LAI-1. In order to maximize the potential effect of LqsA and LAI-1 production on OMV formation, we thought to correlate P_*lqsA*_ activity with OMV formation. In *L*. *pneumophila*, *lqsA* (25) and P_*lqsA*_ (Fig. S4) are expressed from early stationary throughout later stationary growth phase, and therefore, we routinely isolated OMVs from *L. pneumophila* in stationary growth phase.

**Figure 5:**
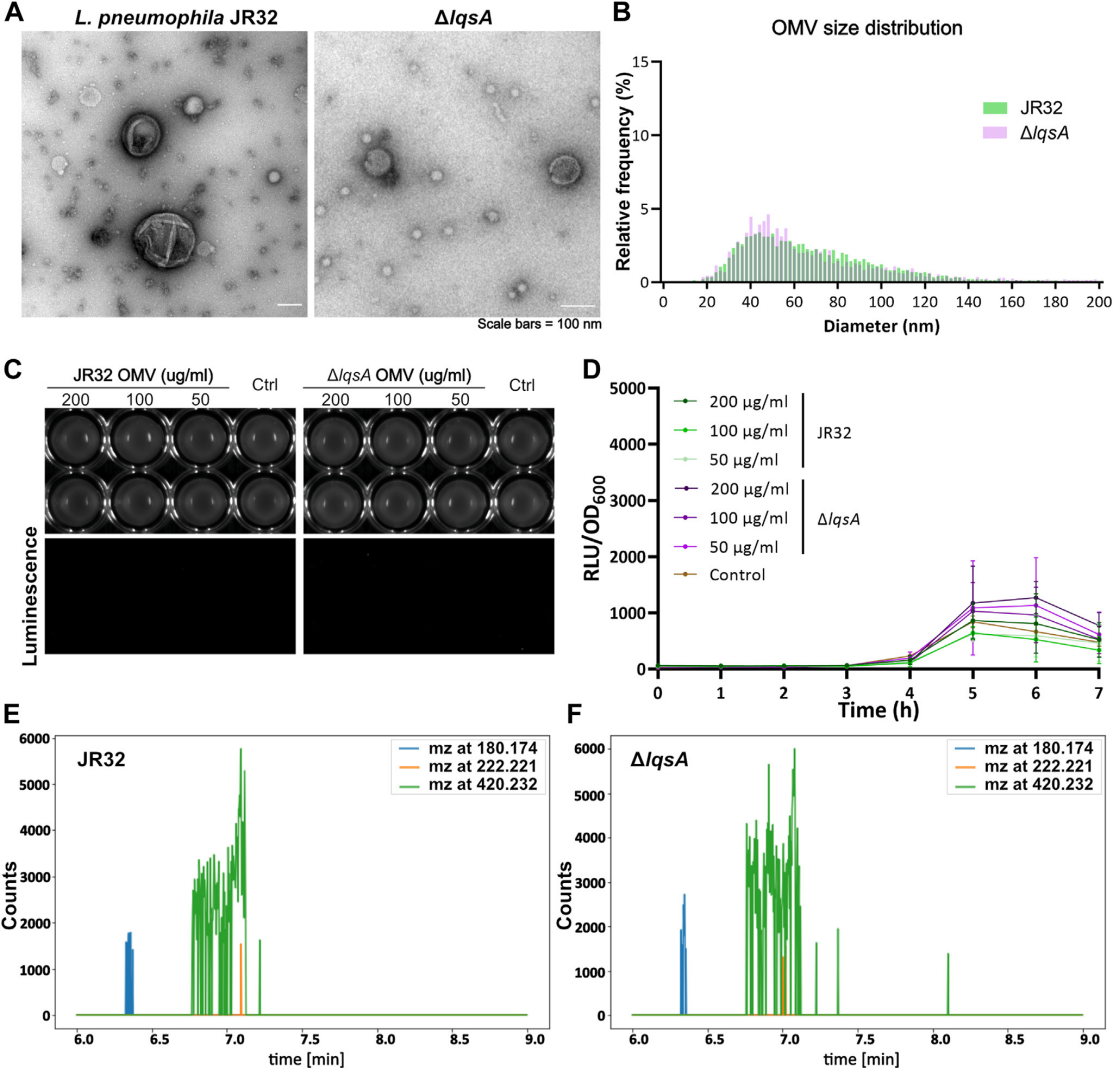
***Legionella pneumophila*strain JR32and Δ*lqsA* produce similar OMV populations.***A*, negative staining TEM images of OMVs derived from *L. pneumophila* JR32 (*left*) or Δ*lqsA* mutant strain (*right*). *B*, size distribution analysis of OMVs from *L. pneumophila* JR32 or Δ*lqsA*. *C* and *D*, luminescence of *Vibrio cholerae* MM920 mixed with OMVs from *L. pneumophila* JR32 or Δ*lqsA* of the protein concentrations indicated (control: DMSO). Intensity was (*C*) visualized by a gel documentation system (15 min exposure time), or (*D*) measured by a plate reader (30 °C, 7 h). *E* and *F*, LC-MS/MS analysis of OMVs of *L. pneumophila* JR32 or Δ*lqsA* (1 mg protein). Extracted ion chromatograms (EICs) of fragment ions at m/z 180.174 (*light blue*), 222.221 (*orange*), and 420.232 (*green*) did not indicate the presence of LAI-1 in these OMVs. The data shown are means and standard deviations of technical triplicates (*D*) and representative of three independent experiments. DMSO, dimethyl sulfoxide; LAI-1, *Legionella* autoinducer-1; LC-MS/MS, liquid chromatography-tandem mass spectrometry; Lqs, *Legionella* quorum sensing; OMVs, outer membrane vesicles; RLU, relative light units; TEM, transmission electron microscopy.

To test whether LqsA affects OMV formation of *L. pneumophila*, we first compared the OMV populations produced by the parental *L. pneumophila* strain JR32 or the Δ*lqsA* mutant strain (Fig. 5*A*). The strains JR32 and Δ*lqsA* produced similar OMV populations, with individual OMVs ranging in size from approximately 20 to 140 nm in diameter and a median diameter of 62 nm (Fig. 5*B*). Hence, under the conditions used, the absence of LqsA does not seem to affect the formation of OMVs in *L. pneumophila*. We then exposed the *V. cholerae* reporter strain MM920 to OMV preparations purified from *L. pneumophila* JR32 or Δ*lqsA* (Fig. 5*C*). Strikingly, none of the OMV preparations tested at a concentration of 50 to 200 μg/ml produced a luminescence signal above the background level of the untreated reporter strain (Fig. 5*D*). Similarly, LAI-1 was also not detected by LC-MS/MS in OMV preparations from strains JR32 (Fig. 5*E*) or Δ*lqsA* (Fig. 5*F*, Table 1). Taken together, under the conditions tested, *L. pneumophila* does not produce and purified OMVs do not contain detectable levels of LAI-1.

**Figure 6:**
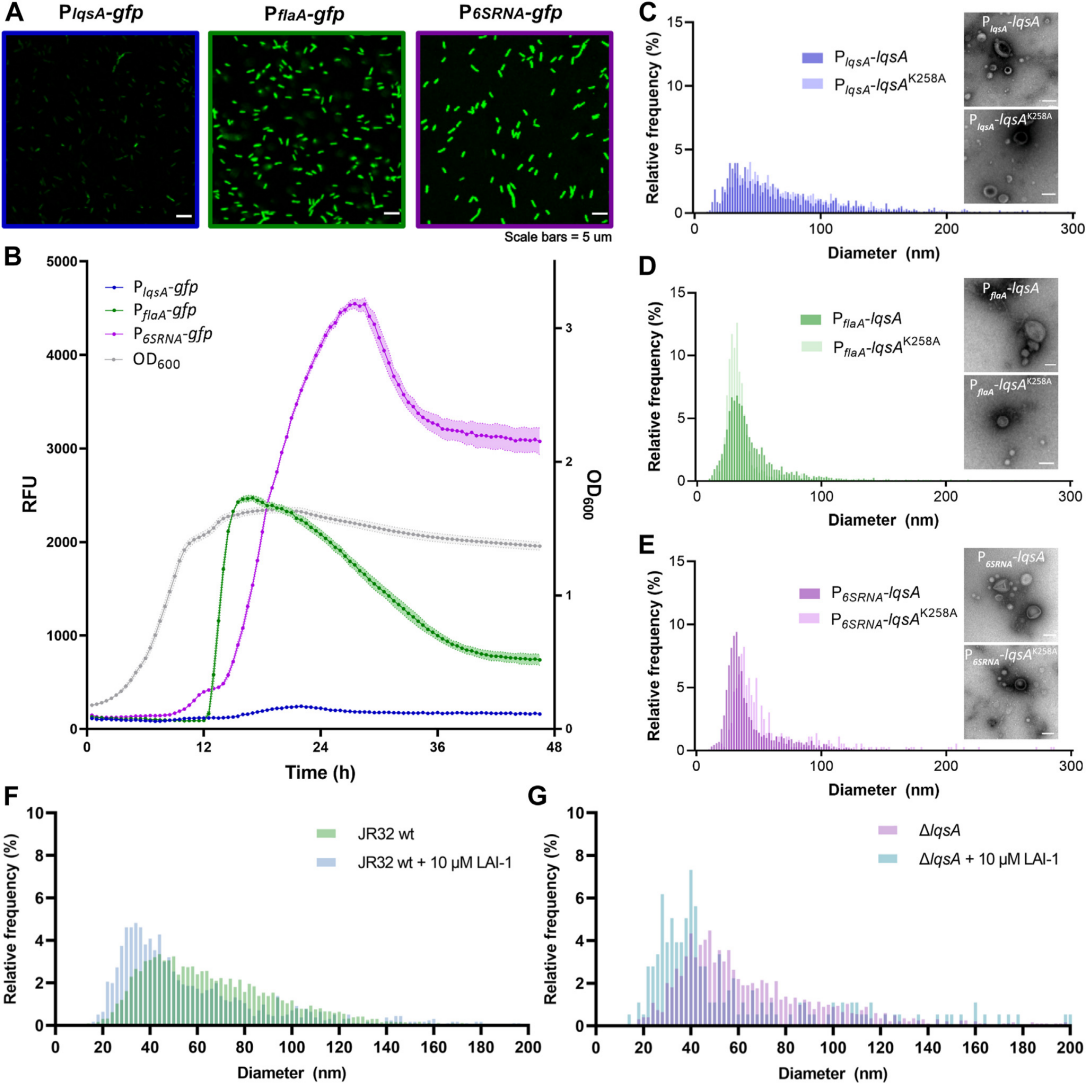
***LqsA* and LAI-1 reduce the median OMV size of *Legionella pneumophila*.** GFP fluorescence intensity was measured by (*A*) confocal fluorescence microscopy of stationary phase *L. pneumophila* JR32 harboring pCM-5 (P_*lqsA*_-*gfp*), pCM-9 (P_*flaA*_-*gfp*) or pRH049 (P_*6SRNA*_-*gfp*), or (*B*) microtiter plate reader of stationary phase cultures diluted to an *A*_600_ of 0.2 (RFU, relative fluorescence units; orbital shaking at 37 °C, 48 h). The intensity of GFP fluorescence indicates that the promoter activity of P_*lqsA*_ is considerably lower than that of P_*flaA*_ or P_*6SRNA*_. *C*–*E*, size distribution and negative staining TEM of OMVs isolated from stationary phase cultures of *L. pneumophila* JR32 harboring (*C*) pAK014 (P_*lqsA*_*-lqsA*) or pTS-24 (P_*lqsA*_*-lqsA*^K258A^), (*D*) pMF04 (P_*flaA*_*-lqsA*) or pMF15 (P_*flaA*_*-lqsA*^K258A^), or (*E*) pMF03 (P_*6SRNA*_*-lqsA*) or pMF16 (P_*6SRNA*_*-lqsA*^K258A^). Strong overexpression of *lqsA* reduces the median OMV size (the scale bars represent 100 nm). *F* and *G*, 10 μM LAI-1 was added to (*F*) *L. pneumophila* JR32 or (*G*) Δ*lqsA* growing in mid-logarithmic phase and the OMV population was assessed. LAI-1 reduces the median OMV size. The data shown are means and standard deviations of technical triplicates (*B*) and representative of three independent experiments. LAI-1, *Legionella* autoinducer-1; Lqs, *Legionella* quorum sensing; OMV, outer membrane vesicle; TEM, transmission electron microscopy.

To increase the levels of LAI-1 produced by *L. pneumophila*, we sought to express *lqsA* from known strong promoters, which initially were tested as transcriptional fusions with *gfp*. Compared to the promoter P_*lqsA*_, the stationary phase promoters P_*flaA*_ and P_*6SRNA*_ indeed led to much higher levels of GFP (Fig. 6*A*). Moreover, judged from the production of GFP, P_*flaA*_ and P_*6SRNA*_ are strongly expressed in the early or later stationary growth phase, respectively, while P_*lqsA*_ is barely expressed (Fig. 6*B*).

Given the features of these promoters, we expressed *lqsA* under the control of P_*flaA*_ or P_*6SRNA*_ and compared the constructs to *lqsA* expressed under the control of its endogenous promoter P_*lqsA*_. The resulting *L*. *pneumophila* strains expressing *lqsA* (WT or a catalytically inactive mutant) under the control of P_*lqsA*_, P_*flaA*_, or P_*6SRNA*_ showed the same morphology and grew indistinguishably in AYE broth (Fig. S5).

*L. pneumophila* expressing *lqsA* under the control of its endogenous promoter P_*lqsA*_ (Fig. 6*C*) produced an OMV population similar to the corresponding strain overproducing a catalytically inactive LqsA mutant. The OMVs ranged in size from approximately 10 to 150 nm diameter. *L. pneumophila* expressing *lqsA* under the control of P_*flaA*_ (Fig. 6*D*) or P_*6SRNA*_ (Fig. 6*E*) produced OMV populations with individual OMV ranging in size from approximately 10 to 100 nm in diameter. The median OMV size was similar in strains expressing *lqsA* or *lqsA*^K258A^ from the same promoter in the case of P_*lqsA*_ and P*_flaA_*: 67 nm/62 nm (P_*lqsA*_), and 37 nm/32 nm (P_*flaA*_). However, the median size of OMVs produced by *L. pneumophila* expressing *lqsA* under the control of P_*6SRNA*_ was significantly smaller than the control (34 nm/45 nm). Overall, the OMVs produced by *L. pneumophila* expressing *lqsA* from its endogenous promoter P_*lqsA*_ were bigger than the OMVs from the strains overproducing LqsA under the control of P_*flaA*_ or P_*6SRNA*_. This was observed independently of the catalytic activity of LqsA, and the reason is unknown.

Based on the finding that overexpression of *lqsA* under the control of P_*6SRNA*_ results in smaller OMVs, we tested whether synthetic LAI-1 affects OMV formation. To this end, we added 10 μM LAI-1 to *L. pneumophila* JR32 or Δ*lqsA* growing in mid-logarithmic phase. Under these conditions, the median size of OMVs from *L. pneumophila* treated with LAI-1 (Fig. 6*F*) was significantly smaller than OMVs from untreated bacteria (Fig. 6*G*): JR32 - 47 nm/62 nm, Δ*lqsA* – 42 nm/58 nm. Therefore, exogenously added synthetic LAI-1 reduced the median OMV size of *L. pneumophila*. Taken together, the *L pneumophila* OMV size distribution and possibly production is affected by the overexpression of *lqsA* or by exogenously added synthetic LAI-1.

### LAI-1 partitions to OMVs of *L. pneumophila* overexpressing *lqsA*

**Figure 7:**
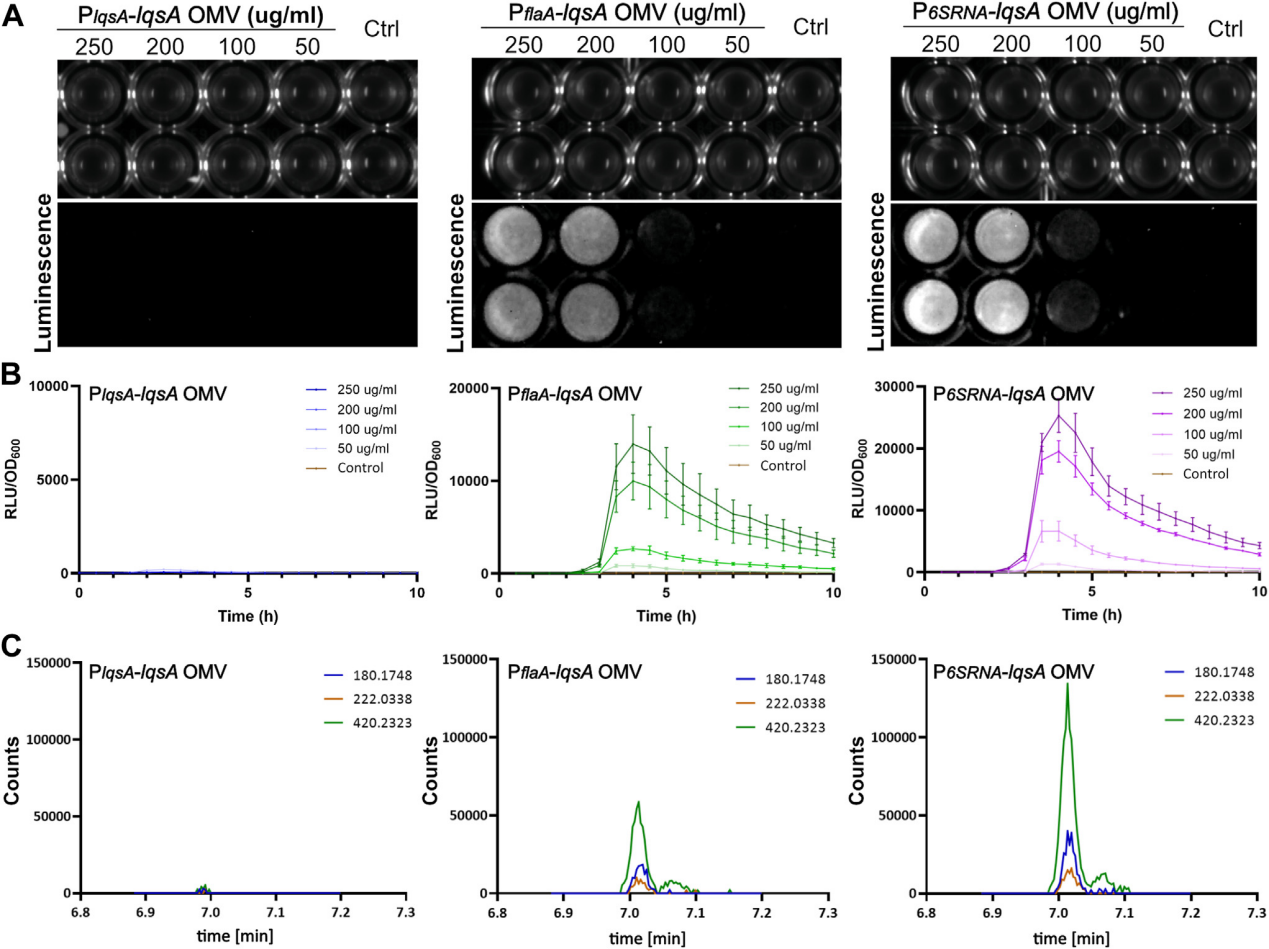
**LAI-1 partitions to OMVs of *Legionella pneumophila* overexpressing *lqsA*.***A* and *B*, luminescence of *Vibrio cholerae* MM920 mixed with OMVs from *L. pneumophila* harboring pAK014 (P_*lqsA*_*-lqsA*), pMF04 (P_*flaA*_*-lqsA*), or pMF03 (P_*6SRNA*_*-lqsA*) of the protein concentrations indicated (control: DMSO). Intensity was (*A*) visualized by a gel documentation system (15 min exposure time), or (*B*) measured by a plate reader (30 °C, 10 h). *C*, LC-MS/MS analysis of OMVs from *L. pneumophila* harboring pAK014 (P_*lqsA*_*-lqsA*), pMF04 (P_*flaA*_*-lqsA*), or pMF03 (P_*6SRNA*_*-lqsA*) (1 mg protein). Extracted ion chromatograms (EICs) of fragment ions at m/z 180.174 (*light blue*), 222.221 (*orange*), and 420.232 (*green*) indicated a significantly higher amount of LAI-1 in OMVs of *L. pneumophila* harboring pMF04 (P_*flaA*_*-lqsA*) or pMF03 (P_*6SRNA*_*-lqsA*). The data shown are means and standard deviations of technical triplicates (*B*) and representative of three independent experiments. DMSO, dimethyl sulfoxide; LAI-1, *Legionella* autoinducer-1; LC-MS/MS, liquid chromatography-tandem mass spectrometry; Lqs, *Legionella* quorum sensing; OMVs, outer membrane vesicles; RLU, relative light units.

To assess whether LAI-1 produced by *L. pneumophila* strains overexpressing *lqsA* from different promoters partitions into OMVs, we exposed the *V. cholerae* reporter strain MM920 to the corresponding OMV preparations (Fig. 7*A*). OMV preparations (50–250 μg protein/ml) purified from *L. pneumophila* strains expressing *lqsA* under control of P_*flaA*_ or P_*6SRNA*_ yielded robust, dose-dependent luminescence signals with a detection limit of ca. 100 μg protein/ml (Fig. 7*B*). In contrast, *L. pneumophila* strains expressing *lqsA* under control of its endogenous promoter P_*lqsA*_ did not elicit any luminescence from the reporter strain. Accordingly, the overexpression of *lqsA* under control of strong promoters produces LAI-1, which partitions to OMVs and activates quorum sensing (luminescence) in a *Vibrio* reporter strain.

Analogous results were obtained upon detection of LAI-1 by LC-MS/MS (Fig. 7*C*, Table 1). LAI-1 was robustly detected and identified in OMV preparations from *L. pneumophila* expressing *lqsA* under control of P_*flaA*_ or P_*6SRNA*_, but barely from an *L. pneumophila* strain expressing *lqsA* under control of P_*lqsA*_. Finally, the expression of a catalytically inactive *lqsA* mutant under control of any of these promoters did not yield LAI-1 in OMV preparations detectable by MS above background levels (Fig. S6, Table 1). In summary, the overproduction in *L. pneumophila* of WT but not catalytically inactive LqsA produces LAI-1, which partitions to OMVs.

### *L. pneumophila* OMVs containing LAI-1 inhibit the migration of *D. discoideum*

**Figure 8:**
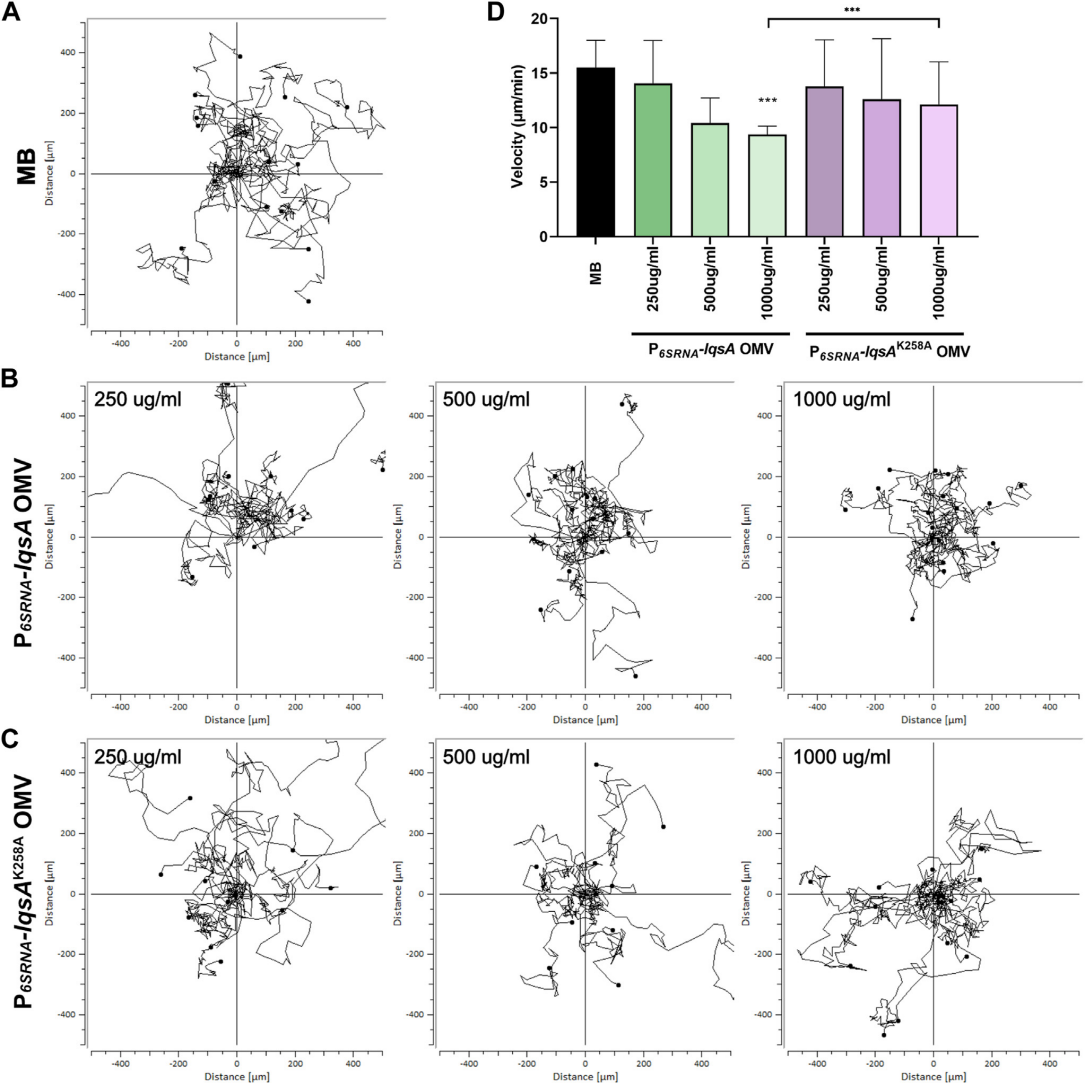
***Legionella pneumophila* OMVs harboring LAI-1 inhibit the migration of amoeba.***Dictyostelium discoideum* amoeba were treated with OMVs of *L. pneumophila* JR32 harboring pMF03 (P_*6SRNA*_*-lqsA*) or pMF16 (P_*6SRNA*_*-lqsA*^K258A^) with a final protein concentration of 250 μg/ml, 500 μg/ml, or 1000 μg/ml. For each sample, the migration of 10 to 15 amoeba was tracked over 2 h and the velocity was quantified. Migration trajectories of amoeba treated with (*A*) (2-(*N*-morpholino)ethanesulfonic acid)-buffered medium (control), (*B*) OMVs of *L. pneumophila* JR32 harboring pMF03 (P_*6SRNA*_*-lqsA*), or (*C*) pMF16 (P_*6SRNA*_*-lqsA*^K258A^). *D*, median of amoeba migration velocity. The data shown are velocity median and standard deviations of 10 to 15 amoeba per sample ∗∗∗*p* ≤ 0.001 and representative of two independent experiments. LAI-1, *Legionella* autoinducer-1; Lqs, *Legionella* quorum sensing; OMVs, outer membrane vesicles.

To address the question, whether *L. pneumophila* OMVs containing LAI-1 affect amoeba migration and thus promote interkingdom signaling, we treated *D. discoideum* with OMVs purified from *L. pneumophila* JR32 producing WT LqsA or the catalytically inactive mutant LqsA^K258A^ under control of P_*6SRNA*_ (Fig. 8). Compared to the MB medium control (Fig. 8*A*), the migration of the amoeba was inhibited in a dose-dependent manner by OMVs purified from *L. pneumophila* expressing WT *lqsA* (Fig. 8*B*), but not by OMVs purified from *L. pneumophila* expressing catalytically inactive *lqsA* (Fig. 8*C*). At the highest OMV concentration used (1000 μg/ml protein), the velocity of the amoeba was reduced ca. 1.7-fold compared to the controls (Fig. 8*D*).

### *LqsA* overexpression promotes intracellular growth of *L. pneumophila*

**Figure 9:**
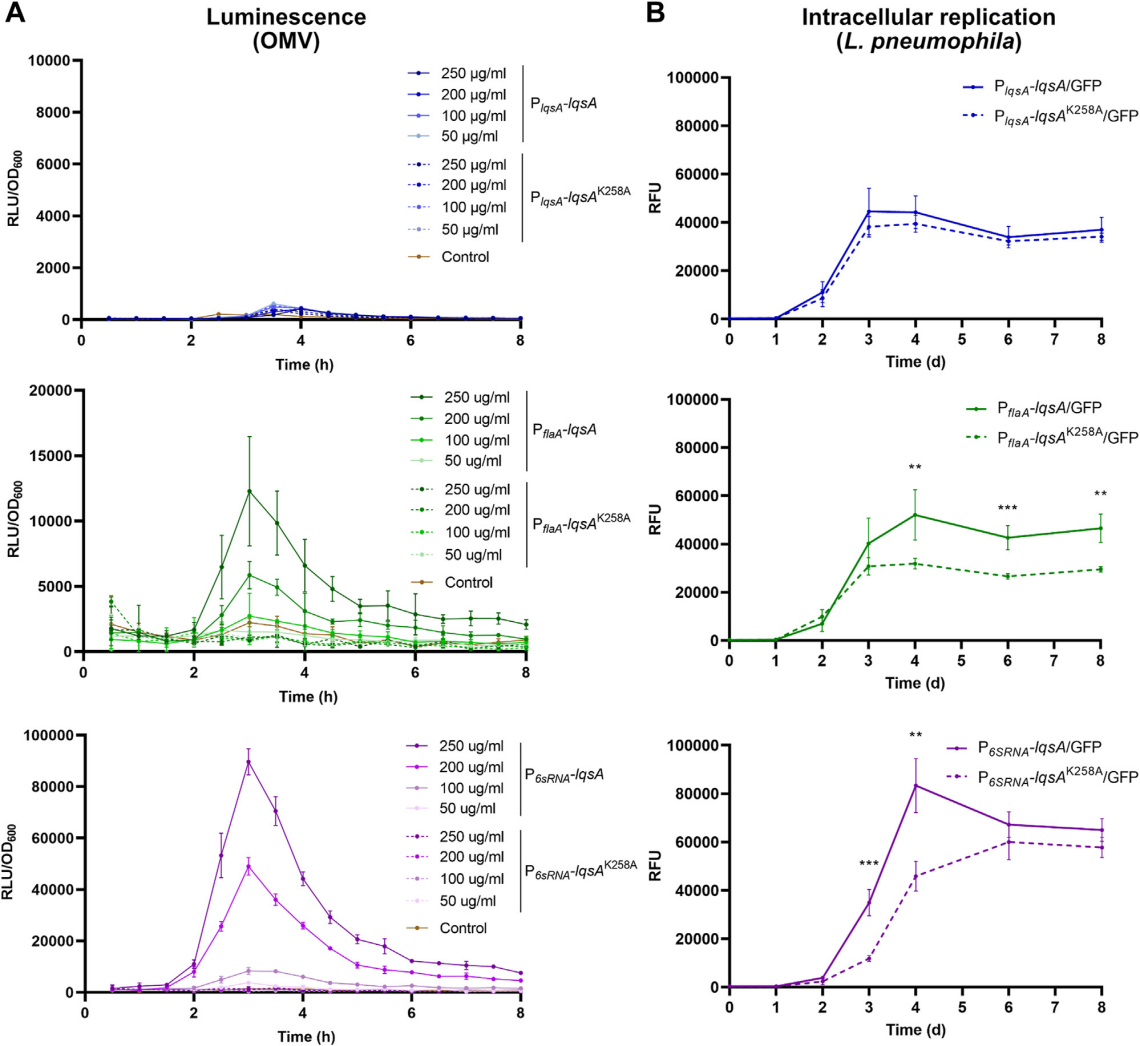
**Overexpression of *lqsA* promotes intracellular replication of *Legionella pneumophila* in macrophages.***A*, OMVs of GFP-producing *L. pneumophila* JR32 harboring pMF21 (P_*lqsA*_*-lqsA*) or pMF22 (P_*lqsA*_*-lqsA*^K258A^), pMF19 (P_*flaA*_*-lqsA*) or pMF20 (P_*flaA*_*-lqsA*^K258A^), or pMF17 (P_*6SRNA*_*-lqsA*) or pMF18 (P_*6SRNA*_*-lqsA*^K258A^) were isolated, and LAI-1 was quantified using the *Vibrio cholerae* reporter strain MM920 (OMV protein concentrations are indicated). *B*, RAW 264.7 macrophages were infected (MOI 5) with GFP-producing *L. pneumophila* JR32 harboring pMF21 (P_*lqsA*_*-lqsA*) or pMF22 (P_*lqsA*_*-lqsA*^K258A^), pMF19 (P_*flaA*_*-lqsA*) or pMF20 (P_*flaA*_*-lqsA*^K258A^), or pMF17 (P_*6SRNA*_*-lqsA*) or pMF18 (P_*6SRNA*_*-lqsA*^K258A^), and intracellular bacterial replication was followed by measuring GFP fluorescence over 8 d. Data shown are means and standard deviations of technical triplicates ∗∗*p* ≤ 0.01; ∗∗∗*p* ≤ 0.001 and representative of three independent experiments. LAI-1, *Legionella* autoinducer-1; Lqs, *Legionella* quorum sensing; OMVs, outer membrane vesicles; RFU, relative fluorescence units.

To test the hypothesis that LAI-1 produced by *L. pneumophila* might affect intracellular pathogen-host interactions, we analyzed the replication in macrophages of *L. pneumophila* strains constitutively producing GFP and LqsA WT or catalytically inactive mutant protein under the control of P_*lqsA*_, P_*flaA*,_ or P_*6SRNA*_. These strains produced GFP at a comparable level (Fig. S7). Using these plasmids producing GFP and LqsA, LAI-1 was detected by the *V. cholerae* reporter strain MM920 in OMVs purified from strains expressing *lqsA* under the control of P_*flaA*_ or P_*6SRNA*_, but not under the control of P_*lqsA*_ (Fig. 9*A*). Intriguingly, upon infection of RAW 264.7 macrophages, the *L. pneumophila* strains overexpressing *lqsA* under the control of the P_*flaA*_ or P_*6SRNA*_ promoters replicated more efficiently in the host cells (Fig. 9*B*). Contrarily, the *L. pneumophila* strain expressing *lqsA* under control of its endogenous P_*lqsA*_ promoter or strains expressing a catalytically inactive *lqsA* mutant did not show enhanced intracellular growth. Hence, the overexpression of *lqsA* but not a catalytically inactive mutant promotes intracellular replication of *L. pneumophila* in macrophages, indicating that intracellularly produced LA1-1 modulates the interaction in favor of the pathogen.

## Discussion

In this study, we analyzed the mechanism of LAI-1 release as well as signaling effects of OMVs produced by *E. coli* and *L. pneumophila* strains expressing the autoinducer synthase gene *lqsA*. We found that the expression of *lqsA* in *E. coli* (Fig. 3) and *L. pneumophila* (Figure 5, Figure 6, Figure 7) produces LAI-1, which partitions to OMVs. The OMV-localizing LAI-1 is bioactive in interbacterial as well as interkingdom signaling. Accordingly, LAI-1 in OMVs produced by *E. coli* triggers luminescence of *V. cholerae* MM920 (Fig. 3, *C* and *D*), induces expression of P_*flaA*_-*gfp* in *L. pneumophila* (Fig. 3*G*), and inhibits migration of *D. discoideum* (Fig. 4). Analogously, LAI-1 in OMVs produced by *L. pneumophila* triggers luminescence of *V. cholerae* MM920 (Fig. 7) and inhibits migration of *D. discoideum* (Fig. 8).

Gene expression analysis by quantitative reverse transcription PCR indicated that *lqsA* is expressed at the onset of the stationary growth phase (25). We observed a similar expression profile for *gfp* under the control of P_*lqsA*_ (Fig. S4). However, compared to the strong stationary promoters P_*flaA*_ and P_*6SRNA*_, the expression of P_*lqsA*_ was very low (Fig. 6). Upon ectopic expression of *lqsA* under the control of different promoters, the strength of promoter activity correlated with the amount of LAI-1 detected by LC-MS/MS in OMVs: P_*6SRNA*_ > P_*flaA*_ > P_*lqsA*_ (Table 1). No LAI-1 was detected in OMVs from *L. pneumophila* producing catalytically inactive LqsA, or in OMVs from *L. pneumophila* JR32 (Table 1). The expression of *lqsA* under control of the P_*tac*_ promoter yielded ca. 30 times less LAI-1 in OMVs from *L. pneumophila* than *E. coli* (Table 1). Taken together, these findings correspond to the observation that *L. pneumophila* strains do not produce LAI-1 detectable by the α-hydroxy-ketone *V. cholerae* reporter strain MM920 or by LC-MS/MS, unless *lqsA* is overexpressed under control of P_*flaA*_ or P_*6SRNA*_ (Figs. 2, 5, 7 and 9). Hence, under the conditions assessed, *L. pneumophila* tightly regulates the production of only low amounts of LAI-1.

*L. pneumophila* might produce higher concentrations of LAI-1 under different physiological conditions. In particular, conditions regulated by the pleiotropic transcription factor LvbR, such as biofilm formation or intracellular growth, could determine *lqsA* expression and LqsA activity. Notably, the production of LAI-1 might be regulated on a transcriptional level (*e.g.*, positive or negative regulation of *lqsA* expression) and/or on a posttranslational level (*e.g.*, covalent attachment of the cofactor pyridoxal-5′-phosphate to LqsA). In any case, the accumulation of LAI-1 in OMVs likely enhances its bioactivity over short distances in highly populated, densely packed biofilms or in the intracellular, organelle-rich milieu of eukaryotic host cells.

The results obtained here with OMV-localizing LAI-1 are similar to results obtained with synthetic LA1-1 for interbacterial signaling (33) and for interkingdom signaling (37). However, OMV-associated LAI-1 and synthetic LAI-1 might be solubilized in different microenvironments, that is, in phospholipid bilayer vesicles or in micelles, respectively. Accordingly, the mode of action (membrane fusion *versus* membrane insertion) and the bioavailability of OMV-associated LAI-1 and synthetic LAI-1 might differ substantially, and consequently, it is challenging to compare the efficacy of the two distinct LAI-1 delivery forms.

While we cannot exclude other transport mechanisms for LAI-1, overall, our findings provide ample evidence that LAI-1 is secreted by OMVs (Figs. 3, 7 and 9). Since LAI-1 is rather hydrophobic, the compound likely partitions into the OMV membrane, but might also be present in the OMV lumen to some extent. As a general concept, packaging in OMVs of hydrophobic, low molecular weight signaling molecules solubilizes these compounds in aqueous environments and facilitates their distribution over rather large distances at biologically active concentrations (42). Hence, by being distributed through OMVs, LAI-1 and other hydrophobic bacterial signaling molecules experience an increased solubility and enhanced bioactivity.

The delivery through OMVs has been shown for a few hydrophobic quorum sensing compounds, which promote bacterial intraspecies and interkingdom signaling (42). The opportunistic human pathogen *Pseudomonas aeruginosa* produces the compound 2-heptyl-3-hydroxy-4-quinolone (*Pseudomonas* quinolone signal), which is packaged into OMVs and mediates quorum sensing-dependent group activities in bacterial populations (52). Intriguingly, *Pseudomonas* quinolone signal stimulates its own OMV packaging and promotes OMV formation of *P. aeruginosa* (52). We obtained evidence that the overexpression of *lqsA* and synthetic LAI-1 modulate the median OMV size of *E. coli* (Fig. 3) and *L. pneumophila* (Fig. 6), and therefore, affect OMV formation. The *Burkholderia thailandensis* hydroxyalkyl quinolone HMNQ is also released through OMVs (53) and *Vibrio harveyi* CAI-1 is packaged in OMVs in late stationary growth phase (54). CAI-1 in *V. harveyi* OMVs triggers quorum sensing phenotypes in CAI-1 nonproducing *V. harveyi* and *V. cholerae*. Moreover, the marine coral pathogen *Vibrio shilonii* produces OMVs that contain quorum sensing molecules of the N-acyl homoserine lactone family (55). Finally, *Paracoccus denitrificans* was shown to release the N-acyl homoserine lactone signaling molecule N-hexadecanoyl-L-homoserine lactone (C16-HSL) *via* OMVs (56).

With regard to interkingdom signaling, LAI-1 is bioactive regardless of whether the compound is delivered extracellularly through purified OMVs from *E. coli* (Fig. 4) or *L. pneumophila* (Fig. 8), or whether the compound is produced intracellularly in host cells infected with *L. pneumophila* overexpressing *lqsA* (Fig. 9). These results are in agreement with the notion that the (unknown) eukaryotic LAI-1 receptor faces the extracellular milieu and, upon uptake of *L. pneumophila*, the lumen of the LCV. This topology would allow the recognition of LAI-1 upon extracellular delivery as well as upon intracellular production. Alternatively, the hydrophobicity of LAI-1 might allow for a fairly unrestricted membrane diffusion and receptor targeting.

The overexpression of *lqsA*, but not a catalytically inactive mutant, promotes intracellular replication of *L. pneumophila* in macrophages (Fig. 9). This result indicates that intracellular—and thus likely continuous or repeated—production of LA1-1 promotes pathogen–host interactions of *L. pneumophila*. Similarly, the overexpression of *lqsA* by intracellular *L. pneumophila* inhibited cell migration of host cells (37). While the inhibition of eukaryotic cell migration could be faithfully recapitulated by the addition of 10 μM synthetic LAI-1, synthetic LAI-1 added prior to or concomitantly with infected macrophages, did not enhance the uptake (37) or intracellular replication of *L. pneumophila*. Perhaps, LAI-1 needs to be produced in a continuous or repeated manner to exert intracellular effects. OMVs shed by intracellular *L. pneumophila* have been reported to inhibit fusion of phagosomes with lysosomes (51) and to promote bacterial replication in macrophages (47). These processes are mechanistically not well understood, but might at least in part rely on LAI-1. Further studies will elucidate the pathways underlying the interkingdom detection of and response to LAI-1 by eukaryotic host cells.

## Experimental procedures

### Bacteria, cells, and reagents

The bacterial strains used in this study are listed in Table S1. *E. coli* TOP10 was cultured overnight at 37 °C in LB broth supplemented with 30 μg/ml Cam. *L. pneumophila* strains were grown for 3 days on charcoal yeast extract agar plates (57), with or without chloramphenicol (Cam; 10 μg/ml) at 37 °C. Bacterial colonies were used to inoculate liquid cultures (starting concentration *A*_600_ of 0.1) in AYE medium (58) at 37 °C on a wheel (80 rpm) and grown for approximately 18 h, with Cam (5 μg/ml) added to maintain plasmids if required.

*V. cholerae* MM920 (22) was cultured overnight at 30 °C in LB broth supplemented with 5 μg/ml tetracycline (Tet) prior to an experiment. *V. cholerae* MM920 lacks the autoinducer synthase gene *cqsA* (Δ*cqsA*) and the sensor kinase gene *luxQ* (Δ*luxQ*), and therefore, does not respond to the quorum sensing signal AI-2, and does not produce but responds to the α-hydroxyketone compounds CAI-1 and LAI-1. Strain MM920 harbors plasmid pBB1, which contains the *luxCDABE* luciferase operon of *V. harveyi* and produces light upon detection of CAI-1 and LAI-1.

Murine macrophage-like RAW 264.7 cells (American Type Culture Collection TIB-71, laboratory collection) were cultivated in RPMI 1640 medium (Life Technologies) supplemented with 10% heat-inactivated fetal calf serum (FCS; Life Technologies) and 1% glutamine (Life Technologies) at 37 °C with 5% CO_2_ in a humidified atmosphere. The macrophages were grown in T75 flasks (Faust Laborbedarf AG) and split every second or third day.

*D. discoideum* strain Ax3 was cultivated in HL5 medium without glucose (Formedium) supplemented with D-maltose (Roth) at 23 °C. The amoebae were grown in T75 flasks and split every third day.

(S)-LAI-1 was synthesized as described (37) and is referred to as “LAI-1” throughout the manuscript.

### Molecular biology and plasmid construction

The plasmids utilized in this study are listed in Table S1. Cloning procedures followed standard protocols, with plasmid isolation performed using commercially available kits from Qiagen. DNA fragments were amplified using Phusion High Fidelity DNA polymerase, using the primers listed in Table S2. Gibson assembly was carried out using the NEBuilder HiFi DNA assembly kit (NEB). All constructed plasmids were validated through Sanger sequencing (Microsynth). To construct plasmid pAK14, P_*lqsA*_-*lqsA* was amplified by PCR using the LqsA-nat-fw and LqsA-mod-rev primers and genomic DNA as a template, and the fragment was cloned into plasmid pTS-28 cut with BamHI and MluI.

The plasmids pMF04 and pMF15 were created by replacing the *gfp* gene in pCM-9 (33) with either the *lqsA* gene or a point-mutated *lqsA*^K258A^ gene, resulting in the transcriptional fusions P_*flaA*_-*lqsA* and P_*flaA*_-*lqsA*^K258A^, respectively. Amplification of the *lqsA* or *lqsA*^K258A^ gene regions was performed using the oMF015 and oMF016 primers, with JR32 genomic DNA or pTS-24 as the respective template. The backbones for pMF04 and pMF15 were identical and obtained by amplifying pCM-9 using the oMF013 and oMF014 primers. Similarly, plasmids pMF03 and pMF16 were constructed as transcriptional P_*6SRNA*_-*lqsA* and P_*6SRNA*_-*lqsA*^K258A^ fusions by replacing the *gfp* gene in pRH049 (36). The *lqsA* or *lqsA*^K258A^ gene regions were amplified using the oMF011 and oMF012 primers, with JR32 genomic DNA or pTS-24 as the respective template. The PCR products were then cloned into pCM-9, amplified by the oMF009/oMF010 primer set.

To construct plasmid pMF17-pMF21, plasmid pNT31 was used as the backbone by digesting with restriction enzyme BamHI. For pMF17 and pMF18, the insert DNA fragments were amplified with the oMF064 and oMF065 primers, using pMF03 and pMF16 as template. The insert DNA fragments of pMF19 and pMF20 were amplified with the oMF066 and oMF065 primers, using pMF04 and pMF15 as templates. The insert DNA fragment of pMF21 was amplified with the oMF067 and oMF065 primers, using JR32 genomic DNA as template. To construct pMF22, plasmid pMF21 was used as the backbone by amplifying with the primers oMF070 and oMF071 primers. Its insert DNA fragment was amplified by oMF069 and oMF065 primers, using pMF16 as template. The amplified DNA insert fragments were cloned into their corresponding backbones using a NEBuilder HiFi DNA assembly kit.

### Isolation of bacterial OMVs

To prepare the bacterial cultures for OMV isolation, *L. pneumophila* strains were grown to late stationary phase (*A*_600_ ∼5.0) in 420 ml AYE medium, supplemented with 5 μg/ml Cam if required. The *E. coli* strains harboring pTS-2 or pTS-10 were cultured in 420 ml of LB medium supplemented with 30 μg/ml Cam. The cultures were maintained at 37 °C with shaking at 120 rpm for 16 h. To induce the expression of *lqsA* in *E. coli* harboring pTS-2 or pTS-10, 1 mM IPTG was added to the bacterial culture, and the incubation was continued for another 5 h.

OMVs were isolated basically as described (54). A low-speed centrifugation step (1500*g*, 15 min, 4 °C) and subsequent membrane filtration (0.45 μm pore size, Huberlab) were carried out to remove bacterial cells and debris from the culture supernatant. The resulting filtrate was then divided equally into six 70 ml polycarbonate bottles (Beckman Coulter) and subjected to ultracentrifugation (150,000*g*, 2 h, 4 °C) using an Optima L-100XP centrifuge with a 45 Ti fixed angle rotor (Beckman Coulter). The resulting pellets were washed with PBS buffer and subjected to another round of ultracentrifugation under the same conditions. This process yielded a clear yellowish solution containing the secreted OMVs. The pellets from the six polycarbonate bottles were resuspended in a total of 1 ml of PBS buffer, and the resuspended samples were filtered once again using 0.45 μm pore size syringe filters. The OMV yield was determined by quantifying their protein content using the Pierce BCA Protein Assay Kit (Thermo Fisher Scientific) according to the manufacturer's instructions. The OMV extracts were either used immediately for experiments or stored at −80 °C for future use, with caution taken to avoid multiple freeze and thaw cycles.

### Gene expression measurements with microplate reader

*L. pneumophila* strains harboring transcriptional *gfp* fusions were grown to late stationary phase (*A*_600_ ∼5.0) in 3 ml AYE medium, supplemented with 5 μg/ml Cam if needed. Each bacterial culture was then diluted with fresh medium to *A*_600_ ∼0.2, and 200 μl of diluted bacterial suspension was pipetted in triplicates in a Falcon 96-well tissue culture-treated microplate (Corning). The growth curve (*A*_600_) and GFP fluorescence intensity (excitation, 488 nm, emission, 528 nm; bottom read) was measured with a BioTek Cytation 5 microplate reader (Agilent Technologies) every 0.5 h for 48 h at 37 °C with continuous orbital shaking.

### LC-MS/MS analysis

For the preparation of MS samples, OMVs extracted from different *L. pneumophila* or *E. coli* strains were subjected to the following procedure. Initially, an OMV sample was combined with dichloromethane in a glass tube at a volume ratio of 2:3. The mixture was then gently agitated on a bench rotator for 1 h at room temperature (RT), with a rotation speed of 4 rpm. Subsequently, the glass tube was allowed to stand undisturbed for 1 h, promoting the separation of the two liquid phases. The organic phase (lower phase) containing the target compounds was carefully collected. To remove the solvent, the collected organic phase was evaporated to dryness using a Concentrator Plus (mode “vacuum – high vapor”, V-HV) for 45 min at RT. The residual solution was reconstituted by adding 150 μl of acetonitrile. The reconstituted sample was then stored at −80 °C until used for analysis.

Prior to analysis, samples were oximated using O-PFB (Merck). To achieve this, a saturated solution of O-PFB in acetonitrile was prepared (ca 100 mg in 500 μl). Ten microliters of derivatization reagent was added to 40 μl sample. Samples were incubated for 10 min at RT prior to analysis. LC separation was performed with a Thermo Ultimate 3000 UHPLC system (Thermo Fisher Scientific) using a C_18_ reversed phase column (Kinetex XB-C18 column, particle size 1.7 μm, pore size 100 Å, dimensions 50 mm × 2.1 mm; Phenomenex). Solvent A was 0.1% (v/v) formic acid and solvent B was 0.1% formic acid in (acetonitrile:H_2_O, 95:5) at a flow rate of 400 μl min^−1^. Solvent B was varied as follows: 0 min, 40%; 2 min, 40%; 5 min, 100%; 7 min, 100%; and 7.5 min, 40%; subsequently, the column was equilibrated for 2.5 min at the initial condition. The injection volume was 2 μl.

MS-product reaction monitoring analysis was carried out with a Thermo Q Exactive plus instrument (Thermo Fisher Scientific) in the positive Fourier transform mass spectrometry. Both experiments were performed with a mass resolution of 17,000 (m/z = 200). In case of product reaction monitoring, a ramped collision energy (20, 25 and 30 eV) was applied. LA1-1 precursor ion at m/z 438.2 (unit resolution) applying high energy C-trap collision dissociation. A heated electrospray ionization probe was used applying following source parameters: vaporizer temperature, 380 °C; sheath gas, 50; aux gas, 20; sheath gas, 50; sweep gas, 0; RF level, 50.0; capillary temperature, 275 °C.

For reproducible estimation of absolute concentrations, external standards with concentrations of 10, 100, 1000, and 8000 nM were measured with each batch. Standard curves were determined from summed peak areas of fragment ions at m/z 180.1748, 222.0338, and 420.2323 whereby fragment ion m/z 180.1748 was used as identification fragment. MS-level 1 analysis was used to confirm exact precursor ion mass.

### *Vibrio* reporter assay

The *V. cholerae* strain MM920 was inoculated in LB liquid medium supplemented with 5 μg/ml Tet and incubated for 18 h at 37 °C. The overnight culture was diluted with fresh medium to an *A*_600_ of 0.25. The medium was supplemented with either synthetic LAI-1 (1–50 μM) or OMV samples (50–250 μg/ml protein concentration).

The mixtures were then transferred to a 96-well plate (Chemie Brunschwig AG) and bioluminescence (luminescence; bottom read) intensity was measured using a Biotek Cytation 5 microplate reader every 0.5 h for 8 to 10 h at 30 °C with continuous orbital shaking. Images were captured after 4 to 5 h incubation (when bioluminescence intensity usually reached maximum levels) using the FluorChem SP imaging system (Alpha InnoTec) with an exposure time of 15 min. All experiments were performed in biological triplicates.

### Single amoeba tracking

*D. discoideum* Ax3 amoeba were seeded at a density of 2 × 10^4^ cells per well into an 8-well Ibidi chamber (Ibidi) and incubated for 3 to 4 h in HL5 medium to allow attachment to the bottom. The medium in each well was then replaced by a diluted OMV sample and incubated at 23 °C for 1 h before microscopy imaging. *E. coli* OMVs were diluted using MB medium (20 mM (2-(*N*-morpholino)ethanesulfonic acid), pH 6.9, 0.7% yeast extract, 1.4% BBL thiotone E peptone) to a final protein concentration of 100 μg/ml, 250 μg/ml, or 500 μg/ml. *L. pneumophila* OMVs were diluted using MB medium to a final protein concentration of 250 μg/ml, 500 μg/ml, or 1000 μg/ml.

### Intracellular replication assay

For infection assays, RAW 264.7 macrophages were seeded at a density of 1 × 10^5^ cells per well onto 24-well tissue culture plates and incubated overnight in RPMI 1640/10% FCS medium. *L. pneumophila* was inoculated at an *A*_600_ 0.2 in AYE medium and grown on a wheel at 37 °C to stationary phase (*A*_600_ ∼5.0, ∼2 × 10^9^ bacteria/ml). The bacterial cultures were then diluted to the desired density in pre-warmed RPMI 1640/10% FCS, and the infection was synchronized by centrifugation (1050*g*, 10 min, RT). After 1 h, the infected cells were washed 4 times with prewarmed RPMI 1640/10% FCS, and further incubated for the time indicated. Depending on the experimental set-up, the infected cells were imaged live, lysed with 0.1% Triton X-100 and/or fixed with 4% paraformaldehyde.

### Transmission electron microscopy

Transmission electron microscopy (TEM) was used for the morphological characterization and size distribution of OMVs extracted from different *L. pneumophila* or *E. coli* strains. TEM was carried out by using a FEI Tecnai G2 Spirit (FEI Company), operating at 120 kV and equipped with a Gatan Orius 1000 (4k × 2.6k pixels) camera (Gatan Inc). FEI was used as the control software, and Gatan DigitalMicrograph was applied for image acquisition.

Prior to TEM, negative staining was performed on OMV samples as follows: A droplet of 5 μl OMV sample (1000 μg/ml diluted with PBS buffer) was added onto a copper a grid (300 square mesh, Formvar Carbon support film; micro to nano) and incubated for 60 s to allow attachment. The liquid was gently blotted away using a Whatman filter paper. A drop of ddH_2_O was then added to wash the grid once. Then a drop of 1% uranyl acetate solution was added onto the grid and incubated for 45 s. After blotting off the liquid, the grid was placed on a filter paper to let dry completely and transferred for TEM.

The size of the OMVs was manually measured from the images acquired by TEM using ImageJ. In each sample, a minimum of 1000 OMVs were individually measured for diameter, and the data were then used to plot the diameter distribution. The average number or concentration of OMVs could not be determined due to technical reasons, since (i) OMVs were nonhomogeneously distributed on the electron microscopy grids, and (ii) the size detection limit of OMVs was <50 nm (smaller OMVs could not be quantified). However, the size distribution and the protein concentration ( ±5%) of OMVs purified from the same bacterial species was very similar, indicating that the overall number of OMVs was also similar.

### Confocal laser scanning microscopy

Fluorescence-based imaging was conducted with a Leica SP8 confocal laser scanning microscope. The following imaging acquisition was used for all imaging experiments: white light laser at 488 nm (2% intensity), fluorescent signal was detected by a power HyD detector (emission range 500–520 nm), transmissive light was detected by a photomultiplier tube detector with a gain of 380. For single amoeba tracking, three fields of interest were randomly selected for each sample and recorded continuously for 2 h with 2 min time interval. Image analyses were performed using ImageJ (https://imagej.net/ij/) and Chemotaxis and Migration Tool version 2.0 (Ibidi).

### Quantification and statistical analysis

Each experiment was independently replicated at least three times, and representative images are shown. All statistical analyses were performed using GraphPad Prism version 7.01 for Windows, GraphPad Software (www.graphpad.com). Two-tailed student’s t tests were used to compare the means of three technical replicates between experimental samples and controls. Significance levels were represented in the figures as follows: ∗, ∗∗, ∗∗∗, or ∗∗∗∗ to indicate probability values of less than 0.05, 0.01, 0.001, and 0.0001, respectively.

## Data availability

All data are contained within the manuscript.

## Supporting information

This article contains supporting information (22, 24, 26, 33, 36, 59, 60).

## Conflict of interest

The authors declare that they have no conflicts of interest with the contents of this article.
