# Supplementary material for: The Legionella autoinducer LAI-1 is delivered by outer membrane vesicles to promote interbacterial and interkingdom signaling
Source: J Biol Chem. 2023 Oct 20;299(12):105376. doi: 10.1016/j.jbc.2023.105376 (PMC10692735; doi:10.1016/j.jbc.2023.105376)
Supplement: Supporting Information [file mmc1.pdf]

Supporting Information

The *Legionella* autoinducer LAI-1 is delivered by outer membrane vesicles to promote interbacterial and interkingdom signaling

Mingzhen Fan[1], Patrick Kiefer[2], Paul Charki[3], Christian Hedberg[4], Jürgen Seibel[3], Julia A. Vorholt[2], and Hubert Hilbi[1]*

[1]*Institute of Medical Microbiology, University of Zürich, Gloriastrasse 30, 8006 Zürich, Switzerland.*

[2]*Institute of Microbiology, ETH Zürich, Vladimir-Prelog-Weg 4, 8093 Zürich, Switzerland.*

[3]*Institute of Organic Chemistry, University of Würzburg, D-97074 Würzburg, Germany.*

[4]*Institute of Chemistry and Umeå Center for Microbial Research, Umeå University, S-90187, Umeå, Sweden.*

Running title: LAI-1 secretion by outer membrane vesicles

***Correspondence:** E-mail hilbi@imm.uzh.ch,

Tel.: +41 (0)44 634 2650, Fax: +41 (0)44 634 4906

Figure S1

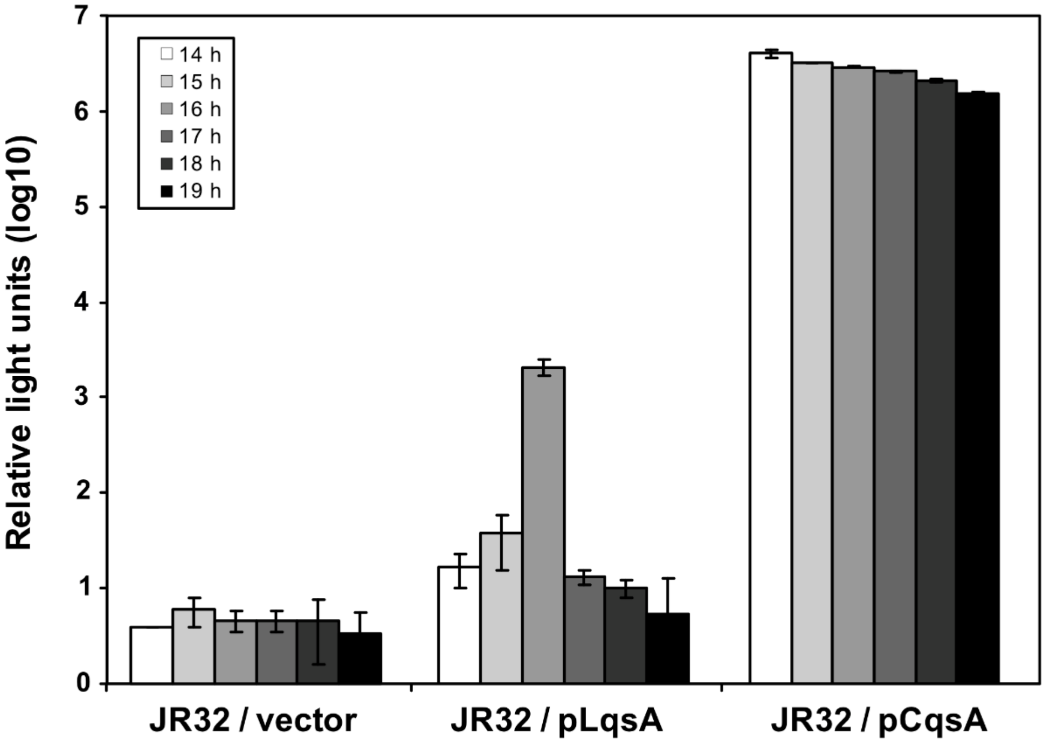

Figure S1. Detection of α-hydroxyketone signal in *L. pneumophila* supernatants by the *V. cholerae* reporter strain MM920. *L. pneumophila* JR32 harboring pTS-10 (vector), pTS-2 (P_{tac}-*lqsA*), or pTS-6 (P_{tac}-*cqsA*) was grown in AYE broth for 14-19 h, and at the time points indicated, α-hydroxyketone activity in the supernatant was assayed by luminescence using the *V. cholerae* reporter strain MM920. The data are means and standard deviations of triplicates.

Figure S2

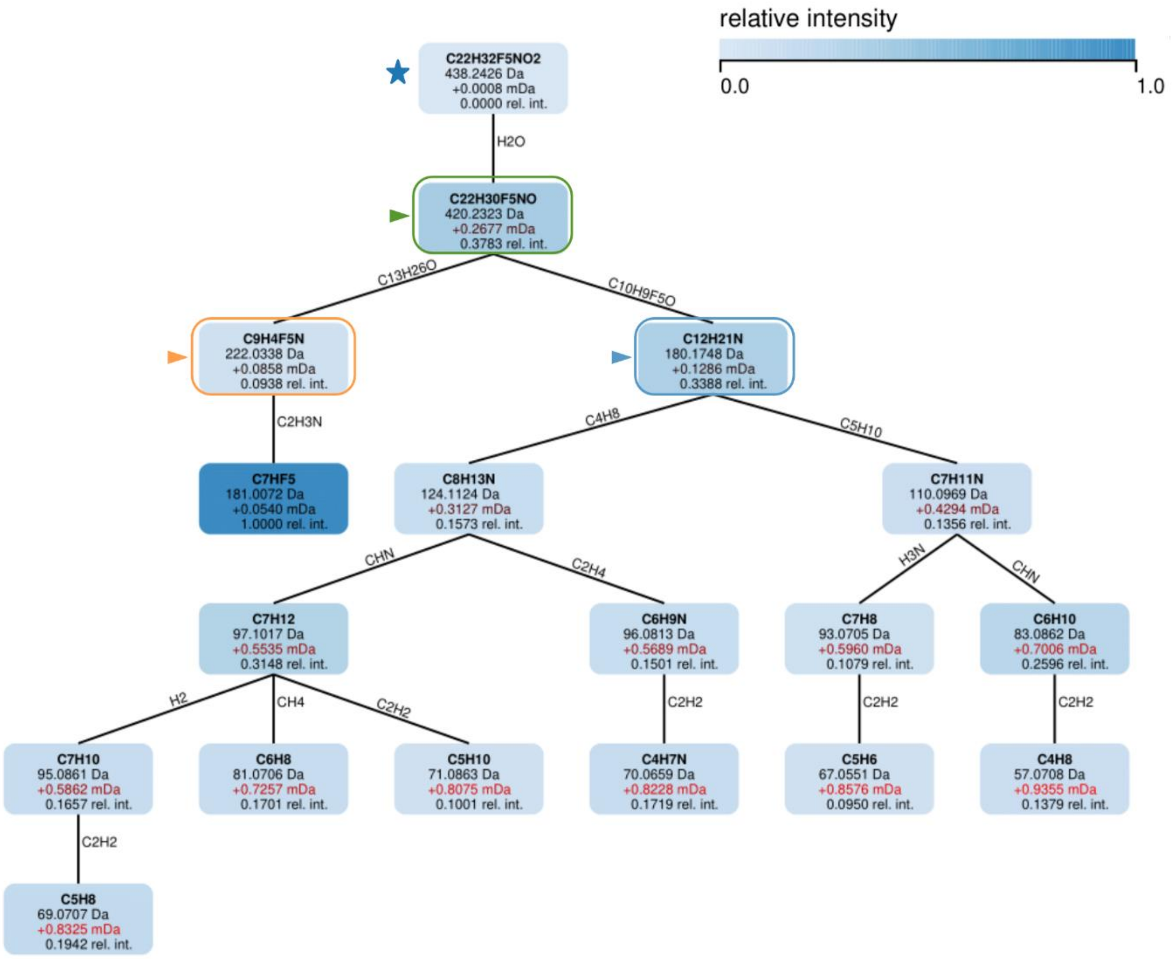

Figure S2. MS fragmentation tree of oximated LAI-1. The complete fragmentation tree of oximated LAI-1 (*Legionella* autoinducer-1, 3-hydroxypentadecane-4-one) is shown, and key fragments are highlighted with a blue star, or green, orange or blue triangles, respectively.

Figure S3

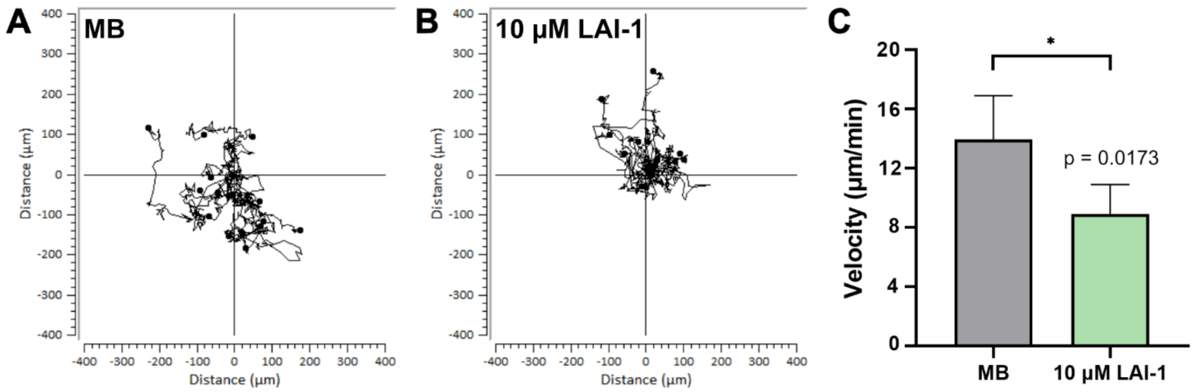

Figure S3. Synthetic LAI-1 inhibits migration of amoeba. *D. discoideum* amoeba were treated with 10 µM synthetic LAI-1. For each sample, the migration of 10-15 amoeba was tracked over 2 h and the velocity was quantified. Migration trajectories of amoeba treated with (**A**) MB medium (control), orn (**B**) 10 µM synthetic LAI-1. (**C**) Median of amoeba migration velocity. The data shown are velocity medians and standard deviations of 10-15 amoeba per sample (*, $p \leq 0.02$) and representative of 3 independent experiments.

Figure S4

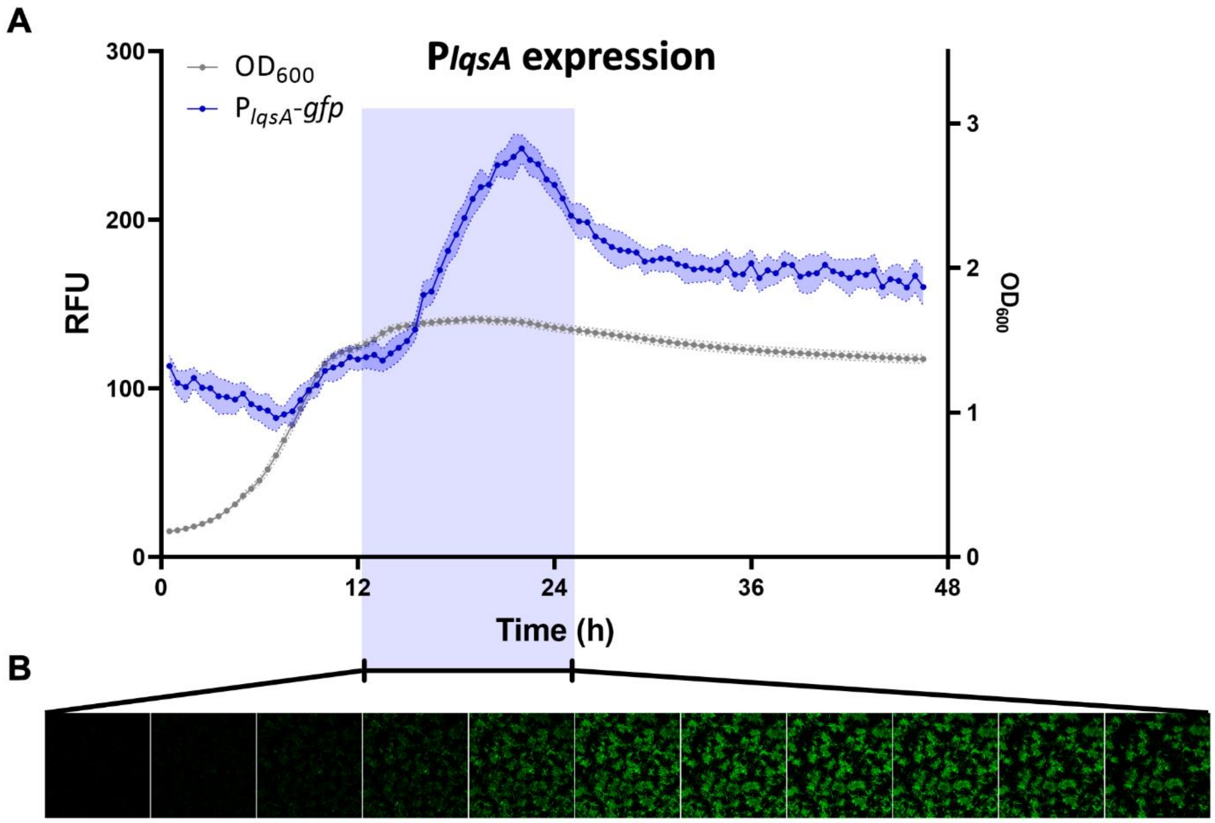

Figure S4. Correlation of *L. pneumophila* growth and *lqsA* expression. (**A**) Stationary phase *L. pneumophila* JR32 harboring pCM-5 (P_{lqsA}-*gfp*) was diluted to an OD_{600} of 0.2 and grown in AYE medium in a 96-well plate while measuring OD_{600} and GFP fluorescence. OMVs were isolated from stationary phase *L. pneumophila*. (**B**) The growth of JR32 harboring pCM-5 (P_{lqsA}-*gfp*) was visualized using confocal microscopy, with each image captured at one-hour intervals to monitor the progressive changes over time. Data shown are means and standard deviations of technical triplicates and representative of 3 independent experiments.

Figure S5

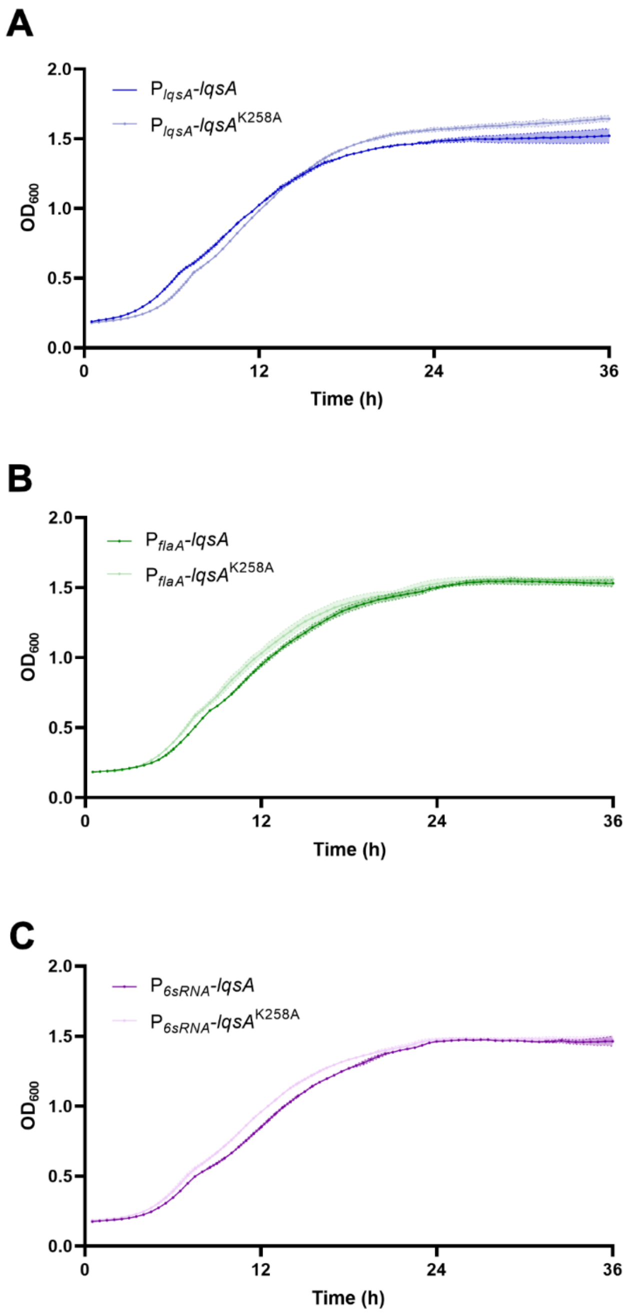

Figure S5. Overexpression *lqsA* does not affect *L. pneumophila* growth in broth. GFP-producing *L. pneumophila* JR32 harboring (**A**) pMF21 (P_{lqsA}-*lqsA*) or pMF22 (P_{lqsA}-*lqsA*^{K258A}), (**B**) pMF19 (P_{flaA}-*lqsA*) or pMF20 (P_{flaA}-*lqsA*^{K258A}), or (**C**) pMF17 (P_{6SRNA}-*lqsA*) or pMF18 (P_{6SRNA}-*lqsA*^{K258A}) were grown in AYE medium (37°C, OD_{600}). Data shown are means and standard deviations of technical triplicates and representative of 3 independent experiments.

Figure S6

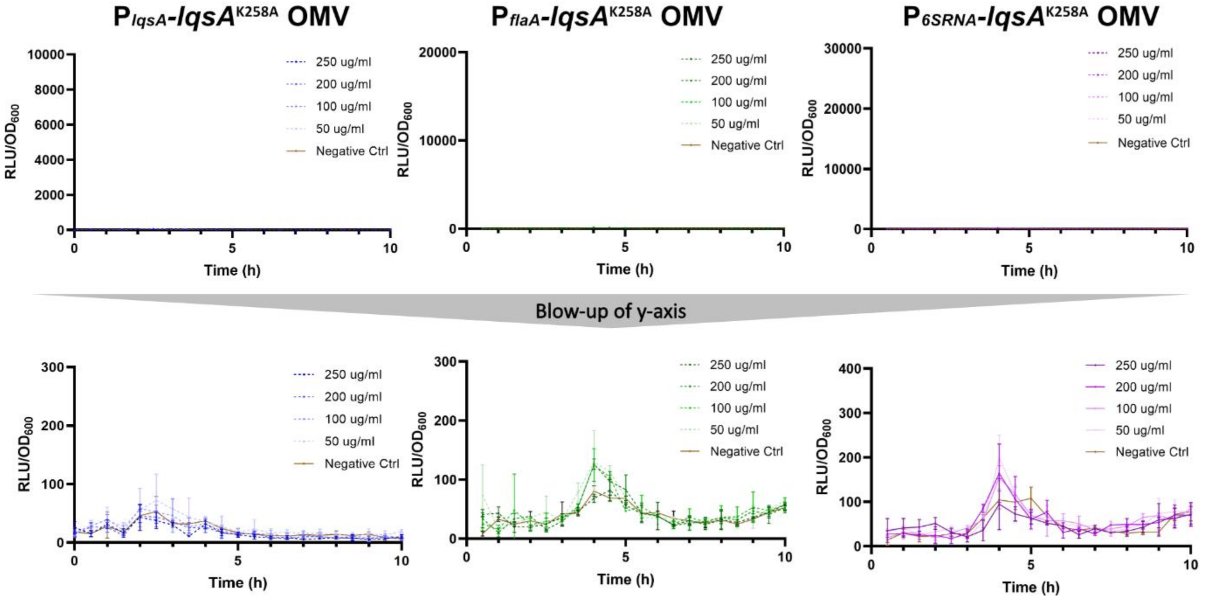

Figure S6. LAI-1 analysis by *V. cholerae* reporter strain of OMVs from *L. pneumophila* expressing catalytically inactive *lqsA*. *V. cholerae* MM920 was treated with OMVs (protein concentrations indicated) from *L. pneumophila* harboring pTS-24 (P_{lqsA}-*lqsA*^{K258A}), pMF15 (P_{flaA}-*lqsA*^{K258A}), or pMF16 (P_{6SRNA}-*lqsA*^{K258A}), and luminescence was measured by a plate reader (30°C, 10 h). RLU, relative light units. Data shown are means and standard deviations of technical triplicates and representative of 3 independent experiments.

Figure S7

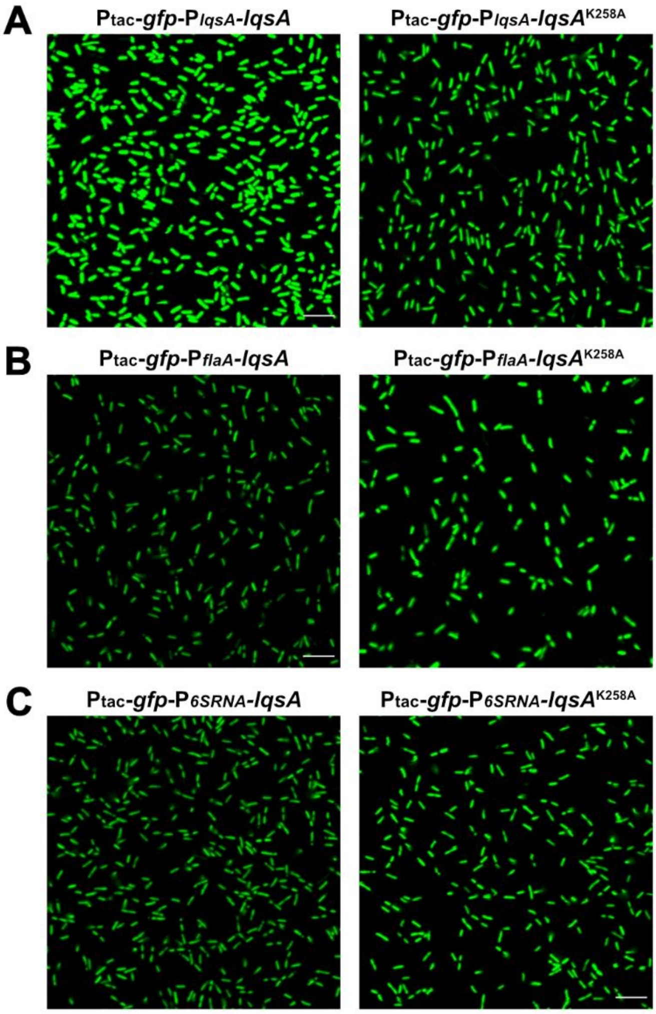

Figure S7. GFP fluorescence intensity of *L. pneumophila* strains overexpressing *lqsA*. GFP-producing *L. pneumophila* JR32 harboring (**A**) pMF21 (P$_{lqsA}$-*lqsA*) or pMF22 (P$_{lqsA}$-*lqsA*^{K258A}), (**B**) pMF19 (P$_{flaA}$-*lqsA*) or pMF20 (P$_{flaA}$-*lqsA*^{K258A}), or (**C**) pMF17 (P$_{6SRNA}$-*lqsA*) or pMF18 (P$_{6SRNA}$-*lqsA*^{K258A}) were grown in AYE medium for 21 h to stationary phase, and fluorescence micrographs were taken by confocal microscopy. Data shown are representative of 3 independent experiments.

Table S1. Bacterial strains and plasmids used in this study.

Strain or plasmid	Relevant properties [a]	Reference
E. coli		
TOP10		Invitrogen
L. pneumophila		
JR32	*L. pneumophila* Philadelphia-1, serogroup 1, salt-sensitive isolate of AM511	(1)
NT02 (Δ*lqsA*)	JR32 *lqsA*::Kan	(2)
Vibrio cholerae		
MM920	ΔcqsA-ΔluxQ, pBB1, Tet (CAI-1/LAI-1 luminescence reporter strain)	(3)
Plasmids		
pAK14	pMMB207C (*lacI*^{q}),P_{lqsA}-*lqsA*, Cam	This study
pCM-5	pMMB207C-P_{lqsA}-*gfp* (ASV) (P_{lqsA}-controlled short-lived GFP-ASV), Cam	(4)
pCM-9	pMMB207C-P_{flaA}-*gfp* (ASV) (P_{flaA}-controlled short-lived GFP-ASV), Cam	(4)
pNT31	pMMB207C, *gfp* (constitutive), *lqsS* (P_{lqsS}), Cam	(2)
pMF03	pMMB207C (*lacI*^{q}), P_{6SRNA}-*lqsA* (P_{6SRNA}-controlled LqsA production)	This study
pMF04	pMMB207C (*lacI*^{q}), P_{flaA}-*lqsA* (P_{flaA}-controlled LqsA production)	This study
pMF15	pMMB207C (*lacI*^{q}), P_{flaA}-*lqsA*^{K258A} (P_{flaA}-controlled LqsA^{K258A} production)	This study
pMF16	pMMB207C (*lacI*^{q}), P_{6SRNA}-*lqsA*^{K258A} (P_{6SRNA}-controlled LqsA^{K258A} production)	This study
pMF17	pMMB207C, P_{tac}-*gfp*, P_{6SRNA}-*lqsA* (P_{6SRNA}-controlled LqsA production)	This study
pMF18	pMMB207C, P_{tac}-*gfp*, P_{6SRNA}-*lqsA*^{K258A} (P_{6SRNA}-controlled LqsA^{K258A} production)	This study
pMF19	pMMB207C, P_{tac}-*gfp*, P_{flaA}-*lqsA* (P_{flaA}-controlled LqsA production)	This study
pMF20	pMMB207C, P_{tac}-*gfp*, P_{flaA}-*lqsA*^{K258A} (P_{flaA}-controlled LqsA^{K258A} production)	This study
pMF21	pMMB207C, P_{tac}-*gfp*, P_{lqsA}-*lqsA* (P_{lqsA}-controlled LqsA production)	This study
pMF22	pMMB207C, P_{tac}-*gfp*, P_{lqsA}-*lqsA*^{K258A} (P_{lqsA}-controlled LqsA^{K258A} production)	This study

pRH049	pMMB207C (*lacI*[q]), P_{6SRNA}-*gfp* (ASV) (P_{6SRNA}-controlled short-lived GFP-ASV), Cam	(5)
pTS-2	pMMB207C, P_{tac}-T7RBS-*lqsA*, Cam	(6)
pTS-6	pMMB207C, P_{tac}-T7RBS-*cqsA*, Cam	(6)
pTS-10	pMMB207C, empty vector, P_{tac}-T7RBS, Cam	(7)
pTS-24	pMMB207C (*lacI*[q]), P_{lqsA}-*lqsA*^{K258A}, Cam	(6)

[a]Abbreviations: Cam, chloramphenicol resistance; Kan, kanamycin resistance; Gen, gentamicin resistance; Tet, tetracycline resistance; RBS, ribosome binding site.

Table S2. Primers used in this study.

Oligo	DNA sequence (5'- 3')
oMF009	GTTTAAGGGTAATTAATAAGCTTGGCTGTTTTGGCG
oMF010	GCTCATATGTATATCTCCTTCTTAAATCTAGACTGGCC
oMF011	GATTTAAGAAGGAGATATACATATGAGCAATACAGAATACCA
oMF012	CCAAAACAGCCAAGCTTATTAATTACCCTTAAACAAAGGA
oMF013	GTTTAAGGGTAATTAAAGCTTGGCTGTTTTGGCGGA
oMF014	GTATTGCTCATAAATTTTAGTCTCCTCAGACCTGAA
oMF015	GTCTGAGGAGACTAAAATTTATGAGCAATACAGAATACCA
oMF016	CGCCAAAACAGCCAAGCTTTAATTACCCTTAAACAAAGGA
oMF064	GATGATCCGTCGATCGCCCGGGGATCCCAGAATTCGAGCTCCATGGCCTGG CTCCT
oMF065	GCATTACGCGTCTCGAGGATCCTTAATTACCCTTAAACAAAGGAATATCGG GTC
oMF066	CTGATGATCCGTCGATCGCCCGGGGATCCCAGAATTCGAGCTCACCAGAAC ATTTAAC
oMF067	CTGATGATCCGTCGATCGCCCGGGGATCCCCCCTTGATTGCAGAAGCGAGT AAATCATC
oMF069	CAAGGGAAAAGAATCTGAGTTATGGATGAGCAATACAGAATACCA
oMF070	GTATTCTGTATTGCTCATCCATAACTCAGATTCTTTTCCCTT
oMF071	CCTTTGTTTAAGGGTAATTAAGGATCCTCGAGACGCGTAATG
LqsA-nat-fw	ATATACGCGTTCCAGAAAGATTTCAATAGGC
LqsA-mod-rev	CGCTGGATCCGTGTTAATTACCCTTAAAC

References

1. Sadosky, A. B., Wiater, L. A., and Shuman, H. A. (1993) Identification of *Legionella pneumophila* genes required for growth within and killing of human macrophages. *Infect Immun* **61**, 5361-5373
2. Tiaden, A., Spirig, T., Sahr, T., Wälti, M. A., Boucke, K., Buchrieser, C., and Hilbi, H. (2010) The autoinducer synthase LqsA and putative sensor kinase LqsS regulate phagocyte interactions, extracellular filaments and a genomic island of *Legionella pneumophila*. *Environ Microbiol* **12**, 1243-1259
3. Miller, M. B., Skorupski, K., Lenz, D. H., Taylor, R. K., and Bassler, B. L. (2002) Parallel quorum sensing systems converge to regulate virulence in *Vibrio cholerae*. *Cell* **110**, 303-314
4. Schell, U., Simon, S., Sahr, T., Hager, D., Albers, M. F., Kessler, A., Fahrnbauer, F., Trauner, D., Hedberg, C., Buchrieser, C., and Hilbi, H. (2016) The alpha-hydroxyketone LAI-1 regulates motility, Lqs-dependent phosphorylation signalling and gene expression of *Legionella pneumophila*. *Mol Microbiol* **99**, 778-793
5. Hochstrasser, R., Michaelis, S., Brülisauer, S., Sura, T., Fan, M., Maass, S., Becher, D., and Hilbi, H. (2022) Migration of *Acanthamoeba* through *Legionella* biofilms is regulated by the bacterial Lqs-LvbR network, effector proteins and the flagellum. *Environ Microbiol* **24**, 3672-3692
6. Spirig, T., Tiaden, A., Kiefer, P., Buchrieser, C., Vorholt, J. A., and Hilbi, H. (2008) The *Legionella* autoinducer synthase LqsA produces an α-hydroxyketone signaling molecule. *J Biol Chem* **283**, 18113-18123
7. Tiaden, A., Spirig, T., Weber, S. S., Brüggemann, H., Bosshard, R., Buchrieser, C., and Hilbi, H. (2007) The *Legionella pneumophila* response regulator LqsR promotes host cell interactions as an element of the virulence regulatory network controlled by RpoS and LetA. *Cell Microbiol* **9**, 2903-2920
